# Patient-directed self-management of pain (PaDSMaP) compared to treatment as usual following total knee replacement; a randomised controlled trial

**DOI:** 10.1186/s12913-018-3146-2

**Published:** 2018-05-10

**Authors:** Katherine H. O. Deane, Richard Gray, Paula Balls, Clare Darrah, Louise Swift, Alan B. Clark, Garry R. Barton, Sophie Morris, Sue Butters, Angela Bullough, Helen Flaherty, Barbara Talbot, Mark Sanders, Simon T. Donell

**Affiliations:** 1 School of Health Sciences, University of East Anglia, Norwich Research Park, Norwich, UK; 2 Norfolk and Norwich University Hospital NHS Foundation Trust, Norwich, UK; 3 Norwich Medical School, Norwich Clinical Trials Unit, University of East Anglia, Norwich, UK; 4 Public & Patient Involvement in Research (PPIRes), South Norfolk Clinical Commissioning Group, Norwich, UK; 5 Norwich Medical School, University of East Anglia, Norwich, UK; 6 Present address: School of Nursing and Midwifery, Latrobe University, Melbourne, Australia; 7 Present address: School of Clinical & Applied Sciences, Leeds Beckett University, Leeds, UK

**Keywords:** Total knee replacement, Pain, Self-medication, Elderly, Randomised controlled trial

## Abstract

**Background:**

Self-administration of medicines by patients whilst in hospital is being increasingly promoted despite little evidence to show the risks and benefits. Pain control after total knee replacement (TKR) is known to be poor.

The aim of the study was to determine if patients operated on with a TKR who self-medicate their oral analgesics in the immediate post-operative period have better pain control than those who receive their pain control by nurse-led drug rounds (Treatment as Usual (TAU)).

**Methods:**

A prospective, parallel design, open-label, randomised controlled trial comparing pain control in patient-directed self-management of pain (PaDSMaP) with nurse control of oral analgesia (TAU) after a TKR. Between July 2011 and March 2013, 144 self-medicating adults were recruited at a secondary care teaching hospital in the UK. TAU patients (*n* = 71) were given medications by a nurse after their TKR. PaDSMaP patients (*n* = 73) took oral medications for analgesia and co-morbidities after two 20 min training sessions reinforced with four booklets. Primary outcome was pain (100 mm visual analogue scale (VAS)) at 3 days following TKR surgery or at discharge (whichever came soonest). Seven patients did not undergo surgery for reasons unrelated to the study and were excluded from the intention-to-treat (ITT) analysis.

**Results:**

ITT analysis did not detect any significant differences between the two groups’ pain scores. A per protocol (but underpowered) analysis of the 60% of patients able to self-medicate found reduced pain compared to the TAU group at day 3/discharge, (VAS -9.9 mm, 95% CI -18.7, − 1.1). One patient in the self-medicating group over-medicated but suffered no harm.

**Conclusion:**

Self-medicating patients did not have better (lower) pain scores compared to the nurse-managed patients following TKR. This cohort of patients were elderly with multiple co-morbidities and may not be the ideal target group for self-medication.

**Trial Registration:**

ISRCTN10868989. Registered 22 March 2012, retrospectively registered.

**Electronic supplementary material:**

The online version of this article (10.1186/s12913-018-3146-2) contains supplementary material, which is available to authorized users.

## Background

The effectiveness of the self-administration of medication (SAM) in the hospital setting has recently been reviewed [1]. It was concluded that “few studies of high methodological quality using validated outcome measures exist. Inconsistencies in both measuring and reporting outcomes across studies make it challenging to compare results and draw substantive conclusions about the effectiveness of SAM schemes”. Following a total knee replacement (TKR) operation, patients experience substantial pain but there is debate as to the best way to manage it [2, 3]. Poor pain control following a TKR can prevent early mobilisation, which substantially raises the risk of patients developing deep-vein thrombosis [4] and can reduce range of motion [5].

This study investigated if a patient’s level of pain and satisfaction with care might be influenced by who is in control of the analgesic medications i.e. whether it is patient-directed self-management of pain (PaDSMaP) or under nurse control (treatment as usual (TAU)). Self-medicating may allow the patient to have more control and take the analgesia “by the clock” [6] but also allow them to vary their analgesia according to pain and activity levels [7].

Traditionally patient controlled analgesia (PCA) has often involved patients being in control of intravenous programmable pumps delivering opioid medications [8]. These have been shown to reduce pain levels by approximately 13 mm (95% CI – 20 to - 6) on a 100 mm VAS at 49–72 h after surgery [8]. They also have very high levels of patient satisfaction [8]. The programmable pump prevents overdoses by locking the patient out after a maximum dose is delivered over a given time period. However the disadvantage of this method of analgesia delivery is that a patient is “tied” to an intravenous pump, and so is less likely to mobilise freely. Additionally the patient is only in control of a single analgesic medication. Optimum post-surgical analgesia requires a combination of analgesics. The addition of non-opioid analgesics (paracetamol, non-steroidal anti-inflammatory drugs, or anti-convulsants such as gabapentin) to an opioid regimen has been shown to improve quality of analgesia and reduce opioid use [9–11].

Our study aimed to investigate whether patients who were in control of all of their oral analgesics experienced lower levels of pain compared to TAU. The primary objective was to investigate if PaDSMaP reduces pain at 3 days or on discharge, whichever is sooner, after primary TKR operation compared to TAU. Secondary objectives investigated whether the PaDSMaP and TAU groups differ in terms of;

- Overall pain levels and pain on mobilization.
- Patient satisfaction with the management of their pain.
- Satisfaction with pain management information provided.
- More global outcomes, such as quality of life (QOL) and activities of daily living (ADLs).
- Time to mobilization, and whether time to mobilization is associated with frequency of adverse events, and improvements in ADLs, QOL and pain at 6 weeks after the operation.
- Incidence of adverse events.
- Quantity and type of pain medications used whilst inpatients.

## Methods

### Study registration

The trial was registered at the Clinical Trials Registry with the International Standard Randomised Controlled Trials Number (ISRCTN10868989) in March 2012. Patient recruitment commenced in August 2011 before registration due to an oversight of the trial team. It had been assumed that it had been registered earlier. The purpose and method of the research were explained to the patients at the pre-admission clinic about 2 weeks prior to their operation when written consent was obtained. The study protocol was published in Trials [12]. The CONSORT checklist is available in Additional file 1.

### Study design and study population

We conducted a prospective, parallel design, open label, randomised controlled, superiority trial of patient-directed self-management of pain (PaDSMaP) compared with nurse control of oral analgesia (TAU) after a total knee replacement [12]. Participants were recruited from the Norfolk & Norwich University Hospital, a secondary care teaching hospital in the United Kingdom.

Competent adults were recruited from patients waiting to undergo a unilateral total knee replacement. The key inclusion criterion was that these patients were able to manage their medications at home. They also had to be English-speaking and literate. We excluded patients that were; opiate-tolerant, opiate-dependent or using modified-release opiate preparations, or had a history of drug or alcohol abuse within the last 5 years. The reasoning for this was that patients who have an established opiate tolerance might be expected to need to continue on an equivalent opiate regimen to prevent opiate withdrawal, and may also require more opiate to ensure equivalent analgesia, and thus would not be comparable to the rest of the population. Other exclusion criteria included expecting post-operative intensive care, or were not competent by reason of dementia or other cause. Post-operatively patients had to be awake and breathing independently, and able to answer questions and follow commands (i.e. competent). Patients were expected to require standard step 1–3 oral analgesics, the 48 patients that received regional blocks or epidural analgesia, started PaDSMaP or TAU as soon as they began oral analgesia. These inclusion and exclusion criteria were in line with the hospital’s drug self-administration policy [13].

The research nurses checked patient eligibility and consent against a checklist. Patients were allocated a personal 6-digit ‘PIN’ identifier that allowed them access to the independent telephone randomisation service at the Clinical Trials Unit (CTU) at the University of East Anglia (UEA). On entry of a valid PIN, the system generated a unique Study Code and randomly allocated the patients to either the PaDSMaP or TAU arm of the trial. The study code and allocation was sent in an email to the research team and stored in the trial database on the secure CTU server at UEA.

A computer generated randomisation list allocated the patients in a 1:1 ratio to PaDSMaP or TAU groups. Randomisation was unstratified. Patients were allocated in randomly distributed blocks of four and six. The patients were randomised, assessed and trained in the pre-operative clinic or at their homes. Patients, their clinical team and the research team were all aware of their allocation. As the intervention was under the control of the patients and the primary outcome (pain VAS) was a patient-reported measure it was impossible to blind patients or clinical staff as to participant’s allocation. It was originally planned to blind the statistician to allocation but as we accidentally included information on duration of self-medication in the statistician’s database they were unblinded, however the analysis plan was pre-specified and approved in a blinded fashion and therefore any potential bias was minimised.

### Study interventions

All participants received the TKR information sheet that is provided as standard to everyone undergoing the operation (Additional file 2). All participants received a study information sheet which informed them that they would be randomised to one of two groups, and in which the two groups’ protocols were described in detail (but this did not include the training pamphlets provided to the self-medicating group once they had been allocated). Care was taken to emphasise the lack of evidence to prefer one protocol over the other. These information sheets are provided in Additional files 3 and 4.

The majority of patients recruited to this study (78%) received the Modified Caledonian Technique (MCT) Norwich Enhanced Recovery Programme (NERP) [14]. This programme aims to enhance the recovery of patients having primary knee replacements by a multi-modal programme, which facilitates early mobility and discharge [15]. The NERP focuses on the provision of safe and effective analgesia with minimal side-effects, which enables early mobility. The analgesia protocol combined a number of classes of analgesics (Table 1). Individual patients were always offered the most suitable protocol for their particular circumstances, so some patients did not follow the NERP protocol. This did not prove an obstacle; as such variations were allowed by our protocol and recorded. Once on the ward TAU patients received their medications as usual from the nurses.

**Table 1:** Range of analgesics provided to patients after the surgery

Analgesic	Usual dose	Notes
**Paracetamol**	**1 g four times a day**	**NERP**
**Ibuprofen**	**400 mg three times a day**	**NERP If patient is able to tolerate**
**Gabapentin**	**300 mg twice a day**	**NERP**
**Oxycodone**	**10-20 mg twice a day**	**NERP doses (Daily EOMD 20–40). Controlled drug so must be delivered by nurse.**
Oxycodone	2.5-10 mg as required	Non-NERP doses. Controlled drug so must be delivered by nurse.
**Oramorph (liquid morphine sulphate)**	**5-20 mg as required up to every 2 h**	**NERP (Daily EOMD 5–240). For breakthrough pain.**
Zomorph (morphine sulphate capsules)	10-20 mg twice a day	(Daily EOMD 20–40)
Codeine	30-60 mg four times a day or as required	(Daily EOMD 18–36)
Dihydrocodeine	30-60 mg four times a day or as required	(Daily EOMD 12–24)
Co-codamol	8/500, 1–2 tablets, once a day or as required 30/500, 2 tablets, four times a day or as required	(Daily EOMD 1.2–2.4) (Daily EOMD 36)
Co-dydramol	10/500, 2 tablets, four times a day	(Daily EOMD 8)
Tramadol	50-100 mg four times a day or as required	(Daily EOMD 20–40)
Aspirin	75 mg once a day	Given as an anticoagulant
**Intramuscular morphine**	**5-10 mg as required**	**NERP (Daily EOMD 5–10). For escape analgesia. Not part of the self-medicating protocol as delivered by injection.**
Intravenous morphine	5-50 mg as required	(Daily EOMD 5–50) Not part of the self-medicating protocol as delivered by injection.
Ropivicaine	0.2% 140-200 ml	Not part of the self-medicating protocol as delivered by periarticular injection.

Bold text rows indicate the medications and dosages recommended for use by the NERP protocol

*Abbreviations*: *NERP* Norwich Enhanced Recovery Protocol, *EOMD* Equivalent Oral Morphine Dose

The PaDSMaP package [12] was developed by members of the research team in conjunction with relevant clinical and lay experts. This resulted in a comprehensive, structured, evidence-based training manual delivered in four pamphlets [3, 16, 17] (Additional file 5). Patients were given these pamphlets in a 20 min training session at the pre-operative clinic outlining why pain control is important, what medications they were likely to be using and how to take them, the common side effects and their management, and non-pharmacological methods for pain relief (e.g. ice packs).

Consultation with lay representatives and healthcare professionals highlighted that it would be inconsistent to allow self-medicating patients to be in control of their oral analgesia but not their oral co-morbid medications. Therefore if patients were prescribed medications for their co-morbidities they were usually in control of these. In general no specific training was provided regarding these medications as patients were expected to have sufficient knowledge to take them appropriately. However patients on anti-hypertensive or anti-coagulant medications were specifically warned to check with nursing staff that it was appropriate for them to take these medications after the operation.

Patients were assessed by a nurse for their competency to manage their own medications, by checking patients were orientated as to location and time, and that they could remember and follow simple instructions [13]. Patients were then given a pack of 14 days-worth of their medications (co-morbid medications and analgesics) previously prepared by the ward pharmacists. All participants received the medications prescribed to them by their clinical team (their consultant, the pain team, and orthopaedic pharmacists).

The UK’s Misuse of Drugs Act (1971) aims to prevent high risk “controlled” medications from being misused, obtained illegally or causing harm. The legislation places legal requirements on the method of prescribing and dispensing of controlled medications. Due to these requirements, any patients prescribed controlled medications (irrespective of their allocation) received them direct from the nurses as they were not approved for self-medication protocols at the hospital [13]. In practice this mostly affected the oxycodone prescribed as part of the standard NERP analgesic protocol. The trial medications were stored in trial-specific lockable bedside cabinets. Patients were given the keys on a lanyard and asked to keep the cabinet locked when not in use and the keys secure. Patients recorded their medication usage on the standard drug chart. This chart had been prepared by the ward pharmacists so that all text was in block capitals, and no abbreviations (e.g. tds) were used. The ward nurses and pharmacists conducted their usual checks of medication usage and record keeping as well as patient competency at the usual drug rounds.

Ward staff (nurses, physiotherapists and the pain team) were trained by the research team about the purpose of the study, the information that the patients would receive and how to assess competency of participants to self-medicate after the operation.

### Baseline measures

At baseline we measured a number of factors in order to provide a description of the characteristics of our sample. Some of these have been identified by other studies as factors that can affect post-operative pain and recovery, and so were possible confounders (Tables 2, 3, 4) [18–23]. Baseline measures were recorded in pre-operative outpatient clinics or in patient’s homes.

**Table 2:** Baseline characteristics

	TAU *n* = 69 unless stated	PaDSMaP *n* = 68 unless stated	All *n* = 137 unless stated
Male	43.5% (30/69)	42.6% (29/68)	41.6 (59/137)
Age (Years)	69.7 (7.5)	70.0 (8.7)	69.8 (8.1)
BMI	32.5 (4.5)	31.5 (5.2)	32.0 (4.9)
Current Smoker	5.9% (4/68)	4.4% (3/68)	5.1% (7/136)
Race	100% Caucasian	100% Caucasian	100% Caucasian
After School Education	55.1% (38/69)	54.4% (37/68)	54.7 (75/137)
Degree education	29.4% (20/68)	32.8% (22/67)	31.1% (42/135)
Employed	15.9% (11/69)	27.9% (19/68)	21.9% (30/137)
Retired	78.3% (54/69)	67.6% (46/68)	73.0% (100/137)
Other	5.8% (4/69)	4.4% (3/68)	5.1% (7/137)
Availability of assistance on discharge?	91.3%(63/69)	86.8%(59/68)	89.1%(122/137)
Presence of stairs at discharge home?	62.3%(43/69)	63.2%(43/68)	62.8%(86/137)
Previous knee op	21.7% (15/69)	22.1% (15/68)	21.9% (30/137)
Previous hip replacement	8.7% (6/69)	7.4% (5/68)	8.0% (11/137)
Duration knee pain (years)^a^	5 (1–66)	3 (1–27)	3 (1–66)
ASA Grade 2 or 3	95.6% (66/69)	95.5% (63/66)	95.6% (129/135)
Other musculoskeletal problems	8.7% (6/69)	26.5% (18/68)	17.5% (24/137)
No. prescriptions^a^	6 (1–17)	6 (0–14)	6 (0–17)
Takes NSAIDS?	52.2% (*n* = 36/69)	59.1% (n = 39/66)	55.6% (75/135)
Number of Comorbidities^a^	3 (0–10)	3 (0–9)	3 (0–10)
% HADS Depressed (≥8) ^a^Median(range)	17.4% (12/69) 3 (0–16)	7.5% (5/67) 3 (0–11)	12.5% (17/136) 3 (0–16)
% HADS Anxious (≥ 8) ^a^Median (range)	14.5% (10/69) 4 (0–13)	17.9% (12/67) 3 (0–13)	16.2% (22/136) 4 (0–13)
NERP protocol implemented	75% (52/69)	82% (54/67)	78% (106/136)

Note: excludes the 7 post randomisation exclusions (but includes 2 withdrawals)

% (x/n) or mean (sd) unless ^a^in which case it was median (range)

*Abbreviations*: *ASA* American Society of Anesthesiologists, *BMI* body mass index, *HADS* Hospital Anxiety and Depression Scale, *NSAIDs* non-steroidal anti-inflammatory drugs, *PaDSMaP* Patient-directed self-management of pain, *TAU* treatment as usual

**Table 3:** Baseline Beliefs about Medicines Questionnaire: subscale totals

Beliefs about Medicines Questionnaire:	TAU *n* = 69 unless stated	PaDSMaP *n* = 68 unless stated	All *n* = 137 unless stated
Specific Needs	17.4 (4.5) *n* = 64	17.8 (5.1) *n* = 63	17.6 (4.8) *n* = 127
Specific Concerns	13.3 (4.1) *n* = 64	11.2 (4.0) *n* = 63	12.3 (4.2) *n* = 127
General Overuse	11.2 (2.6) *n* = 63	10.3 (3.0) *n* = 63	10.7 (2.9) *n* = 126
General Harm	9.7 (2.4) *n* = 63	9.2 (2.7) *n* = 63	9.4 (2.6) *n* = 126

*Abbreviations*: *PaDSMaP* Patient-directed self-management of pain; *TAU* treatment as usual

**Table 4:** Baseline values of outcomes

	TAU *n* = 69 unless stated	PaDSMaP *n* = 68 unless stated	All *n* = 137 unless stated
EQ5D-3 L	0.450 (0.29)	0.463 (0.27)	0.457 (0.28)
Pain VAS (pain over last 4 h)	52.9 (22.3)	48.5 (23.8)	50.8 (23.0)
Oxford Knee Score	18.8 (7.2)	19.0 (7.1)	18.9 (7.1)
Satisfaction with Pain	8.1 (2.5)	8.9 (2.3)	8.5 (2.4)

*Abbreviations*: *EQ5D-3 L* EuroQOL, 3 level; *PaDSMaP* Patient-directed self-management of pain; *TAU* – treatment as usual; *VAS* – pain visual analogue scale

### Outcomes

The primary outcome was pain levels at discharge or after 3 days post-operatively (whichever was the sooner), using a non-graded 100 mm VAS [24]. The pain VAS was anchored by the words “No pain” to “Worst pain” and referred to static pain levels over the last 4 h in the knee that underwent surgery. The primary outcome was measured on the inpatient ward after surgery.

Secondary outcome measures for the patients were:

- Pain levels; static and after mobilisation (3 times a day for days 1–3) and static pain at 6 weeks. Pain VAS were recorded at mealtimes as these were relatively stable in the ward setting; Breakfast 08:00, Lunch 13:00, Supper 17:45. Pain after mobilisation scores related to pain at that moment.
- Satisfaction with pain levels (SWP), medication usage (Days 1–3 and 6 weeks).
- Satisfaction with Information About Medicines Scale (SIMS) [25] (Day 3 or at discharge (if sooner) and 6 weeks).
- Quality of life (using the EQ-5D-3 L) [26, 27], Activities of daily living (using the Oxford Knee Score (OKS)) [28],and a modified version of the client services receipt inventory to assess health-related costs (6 weeks) [29].
- Time to mobilisation was measured from the time the patient returned to the ward after the operation to the time they managed their first transfer out of the bed.
- Adverse events up to 6 weeks were extracted from patient notes.

We planned to investigate medication usage but realised that the high level of variation in the individualised analgesic protocols after surgery (see Table 1) meant that direct comparison between the two groups was impossible.

Secondary measures were measured in the inpatient ward for the 3 days after surgery, and at an outpatient clinic for the 6 week data.

A qualitative evaluation and cost-effectiveness analysis were also undertaken and are reported in detail elsewhere [30].

The qualitative evaluation found that patients had a positive experience of self-medicating even for those patients who reported an initial poor experience of pain control. Some patients reported feeling “isolated” and “a bit concerned I was taking their (nurses) jobs away”. Every patient when asked if they had the option to self-medicate for a similar operation responded positively. Healthcare professionals expressed positive views about the empowering nature of self-medication but had realistic concerns about capacity, documentation and accountability. The paternalistic nature of healthcare was evident (Balls P, Darrah C, Deane KHO, Gray R, Swift L, Barton G, et al: Patient self-management of pain (PaDSMaP); a qualitative investigation of the acceptability of self-medicating with oral analgesics in the context of a randomised controlled trial following total knee replacement, Unpublished qualitative data from this trial).

The cost effectiveness analysis [30] showed that the self-medicating intervention cost £307 to deliver. However most of the costs (£243) were due to set-up costs such as the purchase of bedside cabinets and printing costs. The mean incremental cost for the self-medication group was calculated to be £774 (95%CI £174 to £1374) (when adjusted for age (ns), gender (ns), education (ns) and baseline cost (ns) based on 133 observations, Adj R-squared = 0.0983). However, much of this incremental cost was accounted for by four self-medicating patients who needed intensive care for extended periods of time (for reasons unrelated to the self-mediation protocol). The mean incremental QALY gain was small, 0.002 (95%CI –0.002 to 0.006) (adjusted for age (ns), gender (ns), education (ns) and baseline EQ-5D (*p* < 0.001) based on 133 observations, Adj R-squared = 0.7225). This leads to a mean cost per quality of life adjusted year (QALY) of > £390,000 [30].

### Sample size calculation

Seventy-two patients in each arm was calculated to be sufficient to detect a between-group difference of 0.5 standard deviations in the VAS pain intensity with 80% power using an independent samples t-test. Dahlen [5] reported a standard deviation in VAS pain intensity score of 24 mm 2–5 days after total knee arthroplasty so assuming similar variability this represents a difference of about 12 mm which is within the range of minimum clinically important differences (MCID) that have been reported for pain VAS measures [31, 32]. However, the study was formally powered to detect a difference of 0.5 standard deviations which is therefore the definition of MCID formally used. Calculations used a significance level of 0.05. A drop-out rate of 10% was expected and accounted for.

### Statistical analysis

Linear regression models were used to compare primary and secondary outcomes between the intervention and control. There is no single ‘right’ way of selecting covariates for adjustment. In particular Pocock [33] says that ‘pre-specification of all covariates for adjustment’ while ‘desirable in principle, is often unachievable in practice’ due to ‘inadequate prior knowledge as to which baseline factors are related to prognosis’. This was the case in this study as many factors have been proposed to affect post-operative pain levels [18, 19, 22]. Thus Pocock [33] advocates ‘defining objective variables selection algorithms’ a priori to ‘overcome any suspicions that post hoc selection of covariates might be based on subjective criteria’. Therefore as planned in our original protocol [15], we finalised our analysis plan 6 months before the final data being available, identifying baseline variables which differed between the groups (i) and a set of candidate covariates supplied by the research team (ii) in line with current research [18, 19, 22]. Therefore estimates were also obtained, (i) adjusted by baseline VAS, Hospital Anxiety and Depression Scale (HADS) anxiety and depression scores and (ii) by further potential covariates. (Variables which gave *P* < 0.10 when examined individually as a predictor of the primary outcome, adjusting for variables in (i), were included in a multiple predictor model and then eliminated one by one until all potential predictors had P < 0.10); this resulted in further adjustment by gender and number of prescribed medications. The assumptions of the models were checked using residual analysis which found no outliers or heavily influential observations.

The main analysis was conducted on an ITT basis. For a per protocol analysis successful self-medication was defined as those who started self-medicating and were continuing at 72 h/discharge, even if they stopped for a period during the hospital stay.

Linear regression models were used to investigate, for those randomised to the self-medication group, which baseline variables individually predicted successful self-medication. In order to account for repeated measures, mixed linear regression models were used to compare series of VAS scores measured at breakfast, lunch and supper over the 72 h follow up period, between groups.

In the light of the results, unplanned analyses used the same methods to additionally adjust by day 1 breakfast static VAS scores. SPSS v18 was used for the analysis. The number of co-morbidities was compared between individual included and excluded from the per-protocol analysis using a Mann-Whitney test.

## Results

### Trial flow and baseline characteristics

**Fig. 1:**
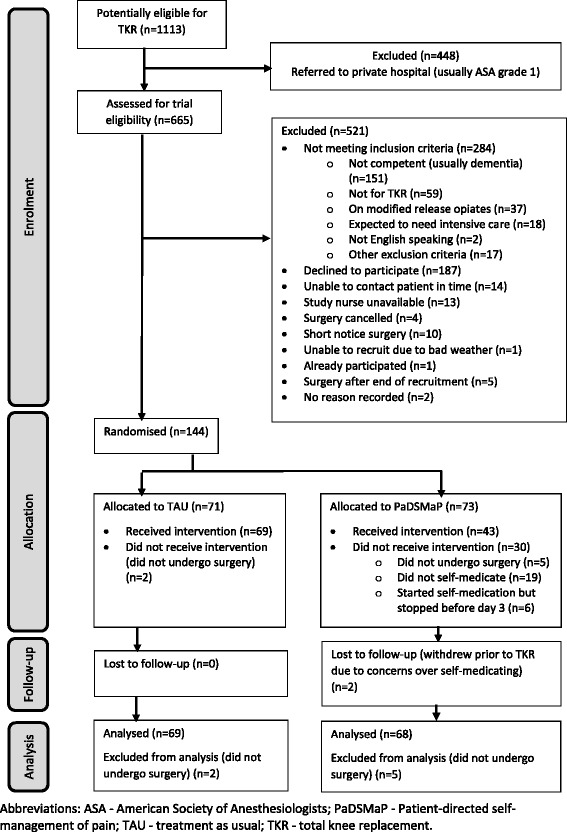
Flow diagram of participants through the trial

Between July 2011 and March 2013, 144 competent adults about to undergo a primary unilateral TKR were recruited (Fig. 1). Patients without co-morbidities (American Society of Anesthesiologists (ASA) grade 1) [34] were generally sent to a private healthcare provider not involved in this study (*n* = 448). Of the 665 patients screened for eligibility for the RCT 23% were excluded because they were not managing their own medications at home (usually due to dementia). A further 28% refused to participate, often because they felt unsure of their ability or desire to self-medicate after a major operation.

Seventy-one patients were randomly allocated to TAU, and 73 to the PaDSMaP group. However seven patients (five PaDSMaP, two TAU) did not undergo surgery for reasons unrelated to the study and were excluded them from the ITT analysis. The remaining patients (*n* = 137) are characterised in Tables 2, 3, 4. They had a median of three co-morbidities in addition to their osteoarthritis. The HADS assessment determined that 13% of patients scored above the cut off for depression, and 17% scored above the cut off for anxiety. Twenty-four patients (18%) had musculoskeletal diagnoses other than osteoarthritis, of which the most common were rheumatoid arthritis (22%) or fibromyalgia (15%).

Half of patients were not taking any opiates prior to their surgery (Table 5). Of those participants prescribed opiates for after their operation, the majority (57 of 68, 84%) were prescribed a single opiate usually to be taken as required (50 of 57, 88%). The maximum prescribed Equivalent Oral Morphine Dose (EOMD) [35] was 96, but most patients were taking substantially lower dosages. It was not possible to calculate the EOMD accurately for each patient due to the variability in the usage of “as required” medication.

**Table 5:** Preoperative opiates

Opiate	Doses	Number of PaDSMaP patients	Number of TAU patients	Maximum daily EOMD
None	–	34	35	–
Codeine	**15 mg as required**	**0**	**1**	
	30-60 mg four times a day	0	2	36
	**30-60 mg as required**	**6**	**3**	
Co-codamol	8/500, 2 tablets, once, twice or three times a day	2	4	7.2
	**8/500 as required**	**1**	**2**	
	**15/500 as required**	**1**	**1**	
	30/500, 2 tablets, once, three or four times a day	3	5	36
	**30/500 as required**	**6**	**12**	
Dihydrocodeine	30 mg once a day	0	1	3
	**30 mg as required**	**1**	**1**	
	60 mg once, twice or four times a day	3	2	24
Co-dydramol	10/500, 1–2 tablets, three or four times a day	1	2	8
	**10/500 as required**	**5**	**6**	
	**30/500 as required**	**0**	**1**	
Tramadol	50 mg once, three or four times a day	0	3	20–40
	**50 mg as required**	**1**	**3**	
	100 mg twice or four times a day	1	1	40–80
	**100 mg as required**	**1**	**3**	
Tramadol paracetamol	**37.5/500, 2 tablets, as required**	**1**	**0**	
Oramorph (liquid morphine sulphate)	**10 mg as required**	**1**	**0**	

Bold text indicate “as required” dosages

*Abbreviations*: *EOMD*, Equivalent Oral Morphine Dose; *PaDSMaP* Patient-directed self-management of pain; *TAU* treatment as usual

The Beliefs about Medicines Questionnaire (BMQ) assesses commonly held beliefs about medicine [21]. The baseline scores (Table 3) show that overall our population agreed that medicines were generally not harmful or overused. The differences between the specific necessity and concerns scores were positive, which indicated that these patients perceive that the benefits of taking medications outweigh the risks.

Of the 68 patients analysed in the PaDSMaP group; 47 started self-medicating (six of whom later stopped (due to losing competency, *n* = 7, or anxiety *n* = 1), and four who started, stopped and then restarted before the end of day 3 (due to losing competency *n* = 3 or anxiety n = 1)), 21 never started (including two who withdrew from the study; due to losing competency up to day 3 *n* = 16 or anxiety *n* = 5), and 41 were classed as having successfully completed the self-medicating protocol (Fig. 1). None of the patients reported any difficulties in opening the medication lockers, and all keys were kept secure for the duration of the study.

Self-medicating patients were also in control of their co-morbid medications. They took a median of 5 (range 0–11) prescriptions. Although at the initiation of the project we were assured that no patients self-medicated on the ward, we found that one diabetic patient allocated to the TAU group did self-medicate with their injectable insulin. All of their remaining medications were delivered by the nurses as usual.

### Baseline comparison

Little difference was observed between the two groups’ baseline characteristics (Tables 2, 3, 4). More of the PaDSMaP group were in employment (28% vs 16%) or had musculoskeletal problems (27% vs 9%), and fewer scored above the cut off for depression (a HADS depression score of ≥8) (7% vs 17%) [20].

### Primary outcome

No evidence was found for a between group difference in ITT mean pain score (static VAS for the previous 4 h) at 72 h/discharge (Table 6). Similar results were obtained after (i) adjustment by baseline VAS, HADS anxiety, HADS depression as specified in the statistical analysis plan and (ii) further adjustment by gender and number of prescribed medications, identified by the covariate selection procedure (Table 6).

**Table 6:** Pain VAS outcomes at 72 h/discharge and 6 weeks

	Outcome	PaDSMaP Mean (sd)	TAU Mean (sd)	Estimated effect^a^ (95% CI)	Difference (95% CI) Adjusted ^b^	Difference (95% CI) Fully adjusted^c^
Intention to Treat analysis	VAS baseline	48.5 (23.8) *n* = 68	52.9 (22.3) *n* = 69			
	VAS static 72 h/discharge	30.3 (21.7) *n* = 64	33.4 (24.0) *n* = 65	−3.1 (− 11.1, 4.9) *p* = 0.441	−3.2 (− 11.5, 5.0) *p*= 0.441	−3.2 (− 11.1, 4.7) *p* = 0.430
	VAS on mobilisation 72 h/discharge	34.6 (24.9) *n* = 60	41.2 (28.9) *n* = 64	− 6.7 (− 16.3,2.9) *p* = 0.170	−6.1 (− 16.2,3.9) *p* = 0.228	−5.7 (− 15.6,4.2) *p* = 0.258
	VAS 6 Weeks	24.4 (19.6) *n* = 65	25.9 (20.6) *n* = 67	− 1.5 (−8.4, 5.5) *p* = 0.676	− 0.87 (−7.9, 6.2) *p* = 0.808	− 0.5 (− 7.5, 6.6) *p* = 0.711
Per protocol analysis^d^	VAS baseline	50.2 (21.8) *n* = 41	52.9 (22.3) *n* = 69			
	VAS static 72 h/discharge	23.5 (18.4) *n* = 40	33.4 (24.0) *n* = 65	−9.9 (− 18.7, − 1.1) *P* = 0.028	−10.3 (− 19.3, − 1.3) *P* = 0.026	−9.6 (− 18.5, − 0.7) *p* = 0.035
	VAS on mobilisation 72 h/discharge	28.2 (21.8) *n* = 39	41.2 (28.9) *n* = 64	− 13.1 (− 23.8,-2.4) *P* = 0.017	−12.1 (− 23.2,-1.2) *P* = 0.030	− 11.5 (− 22.4,-0.6) *P* = 0.039
	VAS 6 Weeks	22.9 (20.5) *n* = 41	25.9 (20.6) *n* = 67	−3.0 (− 11.0, 5.1) *p* = 0.465	−1.7 (− 10.0, 6.7) *P* = 0.693	− 0.8 (− 9.1, 7.5) *P* = 0.849

^a^Estimated treatment effect of self-medication compared to TAU calculated with the linear model (T-test)

^b^T-test adjusted by baseline VAS, HADS anxiety, HADS depression as specified a priori

^c^T-test adjusted by those from a set of variables selected, a priori, with *p* < 0.10 when predicting VAS static, after adjusting by variables in **

^d^Per protocol treatment group is ‘Successful self-medication’ i.e. ‘Started self-medication and continuing at 72 h/discharge even if temporarily stopped during hospital stay’

*Abbreviations*: *HADS* Hospital anxiety and depression scale; *PaDSMaP* Patient-directed self-management of pain; *TAU* treatment as usual; *VAS* visual analogue scale

Using the per protocol definition, patients who were able to self-medicate had significantly lower static VAS (effect − 9.9 mm, 95% CI -18.7 to − 1.1, T-test) and mobilised VAS (effect − 13.1 mm, 95% CI-23.8 to − 2.4, T-test) at 72 h/discharge compared to TAU patients.

### Secondary outcomes

#### Predictors of successful self-medication

Forty-one of 68 patients randomised to self-medicate were ‘successful’ as defined for the per protocol analysis. Only one of 30 baseline variables considered individually showed a significant bivariate association with successful self-medicating. The number of co-morbidities was significantly higher in the group that were unable to self-medicate (mean 3.6 vs 2.7, *p* = 0.007 Wilcoxon Mann Witney).

In an unplanned analysis suggested by the research nurses, lower static pain scores at day 1 breakfast were a highly significant predictor of successful self-medication. (Mean for successful self-medication 44.2 vs 69.2 for those unable to self-medicate, 95% CI of difference 10.9 to 39.2, *P* = 0.001, T-test.)

#### Pain scores

No evidence was found for a between group difference in ITT mean pain after mobilisation score at 72 h/discharge. No significant between group difference was found in pain VAS at 6 weeks (Table 6) whether using ITT or per protocol analysis.

**Fig. 2:**
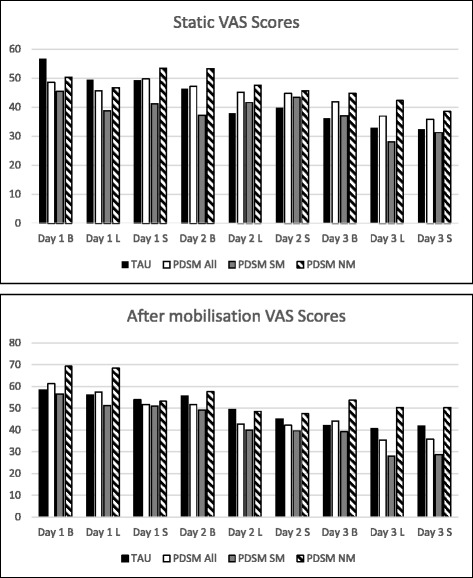
VAS Scores over Days 1–3 After the Operation

In a repeated measures analysis of all nine static post-operative pain scores (breakfast, lunch and supper scores for days 1–3 after the operation, Table 7, Fig. 2) compared between groups, significant day and time-of-day terms suggested that separate models were appropriate at different time points. However, no significant difference was found between the randomised groups for any of the nine time points considered individually.

**Table 7:** VAS Scores over Days 1–3 After the Operation

	Day 1 B	Day 1 L	Day 1 S	Day 2 B	Day 2 L	Day 2 S	Day 3 B	Day 3 L	Day 3 S
Static VAS									
TAU	56.6 (27.5) *N* = 68	49.5 (25.3) *N* = 66	49.3 (25.1) *N* = 62	46.4 (23.6) *N* = 64	37.9 (20.1) *N* = 61	39.8 (25.2) *N* = 58	36.1 (22.4) *N* = 60	32.9 (23.8) *N* = 54	32.3 (23.2) *N* = 43
PaDSMaP	48.6 (31.8) *N* = 60	45.6 (29.5) *N* = 58	49.7 (27.7) *N* = 57	47.1 (26.3) *N* = 59	45.1 (28.5) *N* = 57	44.7 (27.0) *N* = 53	41.8 (26.5) *N* = 58	36.9 (26.6) *N* = 55	35.8 (25.2) *N* = 29
PaDSMaP SM	50.3 (33.4) N = 36	46.7 (33.1) N = 36	53.4 (29.8) *N* = 36	53.3 (26.3) *N* = 35	47.5 (29.3) *N* = 34	45.6 (25.6) *N* = 32	44.8 (27.7) *N* = 35	42.3 (27.3) *N* = 34	38.5 (27.2) *N* = 12
PaDSMaP NM	45.5 (29.1) *N* = 24	38.7 (21.0) *N* = 22	41.2 (21.1) *N* = 21	37.2 (23.3) N = 24	41.6 (26.9) *N* = 23	43.3 (29.1) N = 21	37.1 (23.9) *N* = 23	28.1 (22.8) *N* = 21	31.2 (20.6) *N* = 17
VAS after mobilisation									
TAU	58.5 (28.1) *N* = 51	55.9 (27.9) *N* = 52	53.9 (29.8) *N* = 44	55.6 (26.4) *N* = 58	49.4 (30.3) *N* = 51	45.1 (25.7) N = 41	42.0 (26.3) *N* = 51	40.6 (26.9) *N* = 43	41.8 (29.3) *N* = 33
PaDSMaP	61.2 (27.9) *N* = 57	57.4 (23.2) *N* = 50	51.7 (20.8) *N* = 41	51.7 (23.8) *N* = 56	42.7 (23.8) *N* = 55	42.2 (23.5) *N* = 49	44.2 (24.8) *N* = 58	35.4 (25.0) N = 51	35.8 (25.2) *N* = 41
PaDSMaP SM	56.6 (28.5) N = 36	51.3 (23.1) N = 32	50.9 (21.3) *N* = 26	49.2 (23.2) *N* = 39	39.9 (25.1) *N* = 37	39.6 (25.1) *N* = 33	39.1 (24.5) *N* = 38	27.9 (22.4) *N* = 34	28.7 (20.5) *N* = 26
PaDSMaP NM	69.3 (24.7) *N* = 21	68.3 (19.7) *N* = 18	53.1 (20.0) *N* = 15	57.6 (24.2) N = 17	48.6 (19.4) *N* = 18	47.4 (18.8) *N* = 16	53.7 (22.5) *N* = 20	50.3 (23.1) *N* = 17	50.3 (23.1) *N* = 17

Mean (SD)

*Abbreviations*: *D* Day, *B* breakfast 08:00, *L* lunch 13:00, *S* supper 17:45, *N* number of participants, *SM* self-medicated, *NM* nurse medicated; *PaDSMaP* Patient-directed self-management of pain; *TAU* treatment as usual

An unplanned ‘three group’, repeated measures analysis comparing TAU (*n* = 69), those in PaDSMaP able to self-medicate (*n* = 41), and those in PaDSMaP unable to self-medicate (nurse medicated) (*n* = 27), indicated a particularly large effect between those patients that were unable to self-medicate and TAU on day 1, which reduces by day 3, suggesting again that individual pain time points should be considered separately. The three groups pain scores were significantly different from each other at each of breakfast, lunch and supper of day 1, (*p* = 0.004, 0.009, 0.032 respectively, one-way ANOVA). This led to an exploratory analysis in which we compared the primary outcome, static VAS at 72 h/discharge, between groups, adjusting by day 1 breakfast VAS. This investigates whether group predicts the primary outcome for those with the same pain level at breakfast day 1. In the ITT analysis group this was not significant (− 3.0, 95% CI -10.9 to 5.0 *P* = 0.456, T-test), but day 1 breakfast’s pain score was a strong predictor of the primary outcome (*P* = 0.008, T-test).

#### Other outcomes

No significant between group difference was found in the time to mobilisation (Table 8). Therefore the analyses originally planned to compare time to mobilisation with adverse event frequency, quality of life scores, activities of daily living scores, and level of pain at 6 weeks were not performed. No significant between group difference was found for ED-5D-3 L or OKS at 6 weeks (Table 8). We also found no significant between group difference for satisfaction with pain or information scales at 3 days/discharge or 6 weeks (Table 8).

**Table 8:** ITT group analysis of secondary outcomes

	Outcome	PaDSMaP Mean (sd)	TAU Mean (sd)	Estimated effect^a^ (95% CI) (linear model, T-test)	Difference (95% CI) Adjusted by baseline^b^, HADS anxiety, HADS depression (linear model, T-test)
	Time to Mobilisation	36.1 (23.4) n = 65	34.4 (21.4) n = 66	1.7(− 6.0,9.5) *P* = 0.661	2.8(− 5.1,10.7) *P* = 0.486
Intention to Treat analysis	SWP Baseline	8.87 (2.3) n = 69	8.09 (2.5) n = 68		
	SWP 3 days	9.52 (2.6) n = 53	9.77 (2.7) n = 53	−0.245 (−1.3,0.77) *p* = 0.634	− 0.30 (− 1.36,0.77) *n* = 106 *p* = 0.578
	SWP 6 weeks	10.8 (2.8) n = 63	11.0 (3.2) n = 66	− 0.191 (− 1.2,0.86) *p* = 0.719	−0.25 (− 1.34,0.84) *n* = 127 *p* = 0.647
	SWI 3 days	30.1 (9.6) *n* = 48	31.8 (9.5) n = 54	−1.68 (−5.44,2.08) *p* = 0.379	−1.76 (− 5.58,2.07) *n* = 101 *p* = 0.365
	SWI 6 weeks	28.2 (10.6) n = 61	32.1 (13.5) n = 61	−3.87 (− 8.1,0.64) *p* = 0.093	−4.34 (− 8.87,-0.03) *n* = 120 p = 0.048^c^
	OKS Baseline	19.0 (7.1)	18.8 (7.2)		
	OKS 6 weeks	28.2 (7.2)	27.6 (8.0)	0.55 (−2.1, 3.2) *p* = 0.680	0.486 (−2.1,3.1) *n* = 130 *p* = 0.710

^a^Estimated treatment effect of self-medication compared to TAU

^b^Corresponding baseline outcome or (for SWI), VAS baseline

^c^Anxiety is highly significant here *P* = 0.004 and reduces predicted SWI

*Abbreviations*: *HADS* – Hospital Anxiety and Depression Scale; *ITT* – intention to treat; *OKS* – Oxford Knee Scale; *PaDSMaP* - Patient-directed self-management of pain; *SWP* – Satisfaction With Pain scale; *SWI* – Satisfaction With Information scale; *TAU* – treatment as usual

#### Adverse events

During the study one patient in the self-medicating group took an accidental overdose of morphine sulphate (Oramorph). The patient had started self-medicating at lunchtime on day 1 after surgery. On day 2 at 08:15 the pain team found the patient was a little confused and unsure what medications he had taken. The prescription chart had not been filled in accurately and 30 ml of Oramorph was unaccounted for. After the overdose, Trust policies [13] were followed and it was found that it had resulted in no harm to the patient.

One other patient in the self-medicating group had a medication error. She had brought in her prescribed pregabalin and co-codamol 30/500 from home, and on the morning of day 1 after surgery she started self-medication and took these medications from her home pack. However the patient had been prescribed paracetamol and oxycontin post-operatively. The error was noticed by 08:30 before the patient was given her inpatient pack of medications, and oxycontin was omitted from her morning medication dosage as it was contraindicated with the co-codamol. The patient was reassessed for competency to self-medicate and was assessed as not competent, the medications from home were removed from her locker and she was placed back under the care of the ward nurses for medication delivery which adhered to the hospital prescribed regimen.

Eight patients stated to the nurses that they were anxious about managing to self-medicate after their operation. Five of these patients never started self-medicating, one returned to usual nursing care, one stopped but then re-started self-medicating, one continued self-medicating with support.

There were no medication errors in the TAU group. However over the period of the trial 11 other medication errors occurred on the trial ward in the general patient population of 276, all of whom had nurse controlled medication delivery. These errors included missed medications, incorrect dosage, and incorrect medications. None resulted in any harm to the patients.

Although we recorded a substantial number of other adverse events such as confusion, dizziness, nausea, or vomiting, none of these were determined to be trial-related by our Data Monitoring Committee, which had an independent chair. However these symptoms were the usual reasons given by participants for not self-medicating within the 3 days after surgery that were monitored for the study.

## Discussion

This is the first ever RCT of patient controlled oral analgesia that allowed for patients to self-medicate with individualised poly-pharmacy analgesia protocols and additionally be in control of their co-morbid medications, as far as we are aware [36]. Previous studies have allowed patients to control just one analgesic medication (liquid morphine or short-acting opioids such as morphine or oxycodone) for short periods of time (8–24 h) [37–39]. Our protocol ensured that patients were in charge of all of their analgesics (except for controlled analgesics, as required by law) and co-morbid medications for 3 days after the operation. Our protocol allowed for individualisation of the pain medications prescribed according to patient characteristics, but if the ‘usual’ NERP protocol was followed [14] then our patients would be in control of four different oral analgesic medications (paracetamol, ibuprofen, and gabapentin pills and liquid morphine sulphate), some of which would be new to them. Additionally, in this trial,  these new medications were being taken by medically complex elderly patients. These patients are more representative of modern hospital populations [40] than the more selected patient populations represented in previous RCTs [37–39].

The ITT analysis showed that the pain levels of patients allocated to self-medicate were not superior to those whose medications were dispensed by ward nurses at 3 days/discharge (primary endpoint). Per protocol analysis of the 60% who managed to self-medicate showed that they had reduced levels of pain, however this analysis was underpowered so must be regarded with caution. The size of pain reduction was similar to that shown by meta-analyses of PCA trials [8]. This suggests that the key issue to improve pain levels is who is in control of the analgesia, rather than the method used to facilitate this. It may be that being in control of their analgesia may increase a patient’s psychological resilience and preparedness for painful stimuli. This, in turn, may reduce the emotional component of pain and so make it easier to control [18, 41]. Greater autonomy over pain relief has been linked to improved dignity and patient experience [42, 43]. In turn these factors have been linked to better medication adherence [44].

Greater pain on day 1 after the operation and more co-morbidities predicted those who were unable to self-medicate. This suggests that those with more pain do not start self-medicating or may stop self-medicating. Those patients with higher numbers of co-morbidities may be less well after their operations, thus preventing them from being able to self-medicate.

Since 21% of patients who started self-medicating were unable to continue self-medicating (either temporarily or permanently) within the 3 days after the operation due to confusion, dizziness or vomiting, it highlights the importance of on-going assessment of the patient’s wellbeing and competency to self-medicate by the nurses on their usual drug rounds. Although there are some risks from over-medication, this appears to happen infrequently. As far as we are aware this is the first time an overdose has been reported in an oral self-medicating RCT, with a total of 127 self-medicating patients [37–39]. It should be remembered that nurse-mediated medication delivery is not completely risk free. Thus, as with any medication protocol, risk management is essential. In the case of self-medication it is likely that good patient selection, and training and support of both patients and staff should mitigate this risk to within acceptable levels.

Considering the small pain score differences and the emphasis placed on early mobilisation in the NERP protocol [14], it was unsurprising that the time to mobilisation showed no difference between the two groups. No difference was identified between the groups for quality of life, activities of daily living, satisfaction with information and satisfaction with pain. This may be due to the short duration of impact of the self-medication on global issues such as health-related quality of life or activities of daily living, as these were only measured at baseline and 6 weeks. Both groups received information on their TKR operation, whilst the PaDSMaP group received further information on self-medication, it may be that this was not enough to improve satisfaction with information. Satisfaction with pain has been identified elsewhere [28] as a complex construct that is not solely dependent on pain levels. Both groups were in receipt of individualised pain medication protocols, optimised according to evidence and patient characteristics. So although we detected differences between the groups’ pain levels, it did not change the satisfaction with pain control.

The highly specialist Pharmacists on the ward did prepare the drug packs and drug charts for each patient, and the research nurses conducted the two training sessions. Therefore if this protocol was adopted more generally, workload changes would have to be evaluated.

### Strengths and limitations of this study

The population selected were elderly, had co-morbidities, and the operation required a high level of opiates in order to control pain levels suitably. All of these factors led to a high incidence of adverse events such as confusion, dizziness or vomiting post-operatively, which were the usual reasons for patients not self-medicating. As 40% of patients were unable to complete the self-medicating protocol, the study was effectively under-powered.

The high rate of co-morbidities in our population was due to ASA grade 1 patients (being healthy with a well-controlled co-morbidity [34]) generally being referred to a private healthcare provider which was not involved in the study. This high rate of co-morbidities limits the generalisability of our results to populations with low or no co-morbidities. However our data suggest that these populations would be more likely to manage a self-medicating protocol.

Obviously only patients willing to participate in the trial were recruited. Many of those who refused stated that they were concerned about their ability to manage the perceived complexity of self-medication after an operation. Overall our participants had more positive beliefs regarding the benefits of taking medications when compared to other chronic conditions [21]. Therefore if self-medication was rolled out for all eligible patients, further time might be needed to ensure concerns about the medicines and self-medicating were addressed to patient’s satisfaction.

There was rarely more than one self-medicating patient on the ward at any one time. Sawhney [45] showed that professionals’ knowledge and confidence in self-medicating improved with time and numbers of patients treated. Therefore it is likely that having a greater number of self-medicating patients would have improved understanding and embedded the protocols with ward staff.

There were a few missing pain scores, mostly in the intermediate time points. We know that at least some of these scores were missing due to patients’ unwillingness to complete the research outcomes when they were in severe pain i.e. they were not missing at random. Therefore the intermediate time point’s pain scores may have been under-estimated.

Due to the trial allowing patients to be prescribed individualised analgesia, it is impossible to compare the analgesic “dose” between the groups. Patients were prescribed a range of analgesics. Although the daily Equivalent Oral Morphine Dose (EOMD) could be estimated [35], it would not fairly represent the analgesia provided. Patients usually received additional non-opioid analgesics which have been shown to improve quality of analgesia and reduce opioid use [9–11]. There are currently no methods that allow for the calculation of an overall analgesic dose that includes both opioid and non-opioid analgesics, and even the EOMD estimates are not without controversy and variations in how they are calculated [46].

### Comparison with other studies

As noted in our introduction the evidence base is limited and not consistent. Striebel’s two studies [38, 39] claimed PCOA increased patient satisfaction and pain control compared to patient controlled intravenous analgesia, and Kastanias’ study [37] showed no difference between PCOA and nurse-controlled analgesia on day 2 after a TKR.

### Implications of findings and future research

Currently elective surgical patients have all their inpatient medications controlled by healthcare professionals, yet patients are expected to manage to self-medicate once they are discharged home. For patients capable of self-medication there are potential benefits in terms of improved pain control. However this study demonstrated that self-medication was not suitable for all and a patient’s ability to self-medicate needs to be assessed on an on-going basis. The factors which may limit the ability of adults to self-medicate include having high pain levels on day 1, having higher numbers of co-morbidities, and suffering from adverse events of the surgery and anaesthesia such as confusion and nausea.

Our cost effectiveness analysis [30] showed that the additional costs of self-medication were not large, particularly once set-up costs are accounted for. The calculation of the self-medicating group’s health related costs was complicated by four self-medicating patients requiring periods of time in intensive care (for reasons unrelated to their self-medicating). A longer period of evaluation with a larger cohort would give a clearer picture on the costs of a self-medicating protocol.

We are aware that there are significant political pressures on hospitals to implement self-medicating policies in order to improve patients’ autonomy and dignity [36, 47–50]. The hospital in which this research was conducted now has a target of having all suitable inpatients self-medicating. This study supports this target but highlights that there are significant training needs for both staff and patients. Additionally organisational aspects, such as the availability of Specialist Ward Pharmacists and secure accessible patient lockers, are required to ensure the safe and successful implementation of this policy.

Further research is needed to identify how important this reduction in pain and improvement in autonomy is to patients; whether it is worth the additional training time needed for staff and patients; or if the implementation of self-medication should be for all eligible patients in inpatient settings, and finally, and the evaluation of the health economic implications. The risks of the protocol need to be quantified and management protocols developed that include the development of predictive tools to identify patients unlikely to manage self-medication. Finally the development of a method to calculate the overall analgesic dose for patients prescribed combinations of opioid and non-opioid medications would be helpful.

## Conclusion

This is the first randomised controlled trial of patient controlled oral analgesia that examined individualised multi-medication analgesia. It compared the usual delivery of pharmacological analgesia by nurse-led rounds with patients self-medicating with their drugs held in special locked bed-side cabinets. The self-medicating patients (PaDSMaP) had pain scores that were not superior to the nurse-managed patients (TAU). However 40% of those allocated to self-medication were not able to self-medicate. The 60% that were able to self-medicate had lower pain scores than those managed by nurse-managed care. This cohort of patients were elderly with multiple co-morbidities and may not be the ideal target group for self-medication.

## Additional files

Additional file 1: CONSORT checklist for PaDSMaP Study. (DOC 218 kb)

Additional file 2: Consent for Patients about to undergo Total Knee Replacement Surgery. (PDF 802 kb)

Additional file 3: Information Sheet for Patients in the PaDSMaP Study. (PDF 800 kb)

Additional file 4: Consent form for Patients in the PaDSMaP Study. (PDF 741 kb)

Additional file 5: Training pamphlets for the PaDSMaP study. (PDF 1589 kb)

## Abbreviations

ASA: American Society of Anesthesiologists
B: breakfast 08:00
BMI: body mass index
D: Day
EOMD: Equivalent Oral Morphine Dose
EQ5D-3 L: EuroQOL 3 level
HADS: Hospital Anxiety and Depression Scale
L: lunch 13:00
N: Number of participants
NERP: Norwich Enhanced Recovery Protocol
NM: Nurse medicated
NSAIDs: Non-steroidal anti-inflammatory drugs
PaDSMaP: Patient-directed self-management of pain
PDSM All: PaDSMaP self-medicating all patients allocated to group
PDSM NM: PaDSMaP self-medicating patients that were nurse medicated
PDSM SM: PaDSMaP self-medicating patients that self-medicated
S: Supper 17:45
SM: Self-medicated
TAU: Treatment as usual
TKR: Total knee replacement
VAS: Pain visual analogue scale
