# Supplementary material for: Patient-directed self-management of pain (PaDSMaP) compared to treatment as usual following total knee replacement; a randomised controlled trial
Source: BMC Health Serv Res. 2018 May 10;18:346. doi: 10.1186/s12913-018-3146-2 (PMC5944138; doi:10.1186/s12913-018-3146-2)
Supplement: Supplementary file 1 — CONSORT checklist for PaDSMaP Study. (DOC 218 kb) [file 12913_2018_3146_MOESM1_ESM.pdf]

Insert Patient ID label

Consent form 1
Patient agreement to: TOTAL KNEE REPLACEMENT

Patient details (or pre-printed label)
Patient's surname/family name..
Patient's first names ....
Date of birth ....
Responsible health professional..
Job title ....
NHS number (or other identifier)..
I Male I Female
Special requirements ....
(eg other language/other communication method)

To be retained in patient's notes

Name of proposed procedure or course of treatment:
TOTAL KNEE REPLACEMENT LEFT / RIGHT SIDE (delete as appropriate)

Statement of health professional (to be filled in by health professional with appropriate knowledge of proposed procedure, as specified in consent policy).
I have explained the procedure to the patient. In particular, I have explained:

The intended benefits:
Pain relief, improved mobility

Serious or frequently occurring risks:
☐ Infection
☐ Venous thromboembolism (blood clots)
☐ Nerve or vessel damage
☐ Fractures
☐ Mechanical sensations/noises from components
☐ Implant loosening and wear requiring revision surgery
☐ General complications such as chest infection, heart attack, stroke and death

Please tick when discussed with patient

Any extra procedures which may become necessary during the procedure

☐ blood transfusion ☐ other procedure (*please list below)*

I have also discussed what the procedure is likely to involve, the benefits and risks of any available alternative treatments (including no treatment) and any particular concerns of this patient.

☐ The following leaflet has been provided (**please specify name below)*

………………………………………………………………………………………………

This procedure will involve:

☐ general and/or regional anaesthesia ☐ local anaesthesia ☐ sedation

Signed:………………………………………… Date .. ……………………..……….

Name (PRINT) ………………………. ……… Job title …….. ……………………..…

Contact details (if patient wishes to discuss options later) …..………………..……………

Statement of interpreter (where appropriate)

I have interpreted the information above to the patient to the best of my ability and in a way in which I believe s/he can understand.

Signed ……………………..……………………. Date ………………..…………….

Name (PRINT) ………………..……………………………………………………………

Statement of patient

Please read this form carefully. If your treatment has been planned in advance, you should already have your own copy of this form which describes the benefits and risks of the proposed treatment. If not, you will be offered a copy now. If you have any further questions, do ask – we are here to help you. You have the right to change your mind at any time, including after you have signed this form.

I agree to the procedure or course of treatment described on this form.

I understand that you cannot give me a guarantee that a particular person will perform the procedure. The person will, however, have appropriate experience.

I understand that I will have the opportunity to discuss the details of anaesthesia with an anaesthetist before the procedure, unless the urgency of my situation prevents this. (This only applies to patients having general or regional anaesthesia.)

I understand that any procedure in addition to those described on this form will only be carried out if it is necessary to save my life or to prevent serious harm to my health.

Insert Patient ID label

I have been told about additional procedures which may become necessary during my treatment. I have listed below any procedures **which I do not wish to be carried out** without further discussion.
…………………………………………………………………………

I agree to the use of photography for the purpose of diagnosis and treatment

I agree photographs and x-rays being used for medical teaching

National Joint Registry: I have read and comprehended the information sheet supplied and would like my details to be submitted to the National Joint Registry in the full knowledge that they will only be disclosed by law or for an overriding public interest. I have been assured that by declining my consent my care and treatment would not be affected in any way.

Patient's signature ………………………………………….. Date…………………………..
Name (PRINT)
………………………………………………………………………………………

A witness should sign below if the patient is unable to sign but has indicated his or her consent. Young people/children may also like a parent to sign here (see notes).

Signature …………………………………………… Date ……………………..…..………
Name (PRINT) ……………………………………………………………………………..…

Confirmation of consent (to be completed by a health professional when the patient is admitted for the procedure, if the patient has signed the form in advance). On behalf of the team treating the patient, I have confirmed with the patient that s/he has no further questions and wishes the procedure to go ahead.

Signed:…….…………………………………… Date .. …………………….………..
Name (PRINT) ………………………. ……… Job title ……... ……………………..…

Important notes: (tick if applicable)

- ☐ See also advance directive/living will (eg Jehovah's Witness form)
- ☐ Patient has withdrawn consent (ask patient to sign /date below)

……………..………………………………………………………………………….

Insert Patient ID label

Guidance to health professionals (to be read in conjunction with consent policy)

What a consent form is for
This form documents the patient's agreement to go ahead with the investigation or treatment you have proposed. It is not a legal waiver – if patients, for example, do not receive enough information on which to base their decision, then the consent may not be valid, even though the form has been signed. Patients are also entitled to change their mind after signing the form, if they retain capacity to do so. The form should act as an *aide-memoire* to health professionals and patients, by providing a check-list of the kind of information patients should be offered, and by enabling the patient to have a written record of the main points discussed.

In no way, however, should the written information provided for the patient be regarded as a substitute for face-to-face discussions with the patient.

The law on consent
See the Department of Health's *Reference guide to consent for examination or treatment* for a comprehensive summary of the law on consent (also available at www.doh.gov.uk/consent).

Who can give consent
Everyone aged 16 or more is presumed to be competent to give consent for themselves, unless the opposite is demonstrated. If a child under the age of 16 has "sufficient understanding and intelligence to enable him or her to understand fully what is proposed", then he or she will be competent to give consent for himself or herself. Young people aged 16 and 17, and legally 'competent' younger children, may therefore sign this form for themselves, but may like a parent to countersign as well. If the child is not able to give consent for himself or herself, some-one with parental responsibility may do so on their behalf and a separate form is available for this purpose. Even where a child is able to give consent for himself or herself, you should always involve those with parental responsibility in the child's care, unless the child specifically asks you not to do so. If a patient is mentally competent to give consent but is physically unable to sign a form, you should complete this form as usual, and ask an independent witness to confirm that the patient has given consent orally or non-verbally.

When NOT to use this form
If the patient is 18 or over and is not legally competent to give consent, you should use form 4 (form for adults who are unable to consent to investigation or treatment) instead of this form. A patient will not be legally competent to give consent if:

- they are unable to comprehend and retain information material to the decision and/or
- they are unable to weigh and use this information in coming to a decision.

You should always take all reasonable steps (for example involving more specialist colleagues) to support a patient in making their own decision, before concluding that they are unable to do so.
Relatives **cannot** be asked to sign this form on behalf of an adult who is not legally competent to consent for himself or herself.

Information
Information about what the treatment will involve, its benefits and risks (including side-effects and complications) and the alternatives to the particular procedure proposed, is crucial for patients when making up their minds. The courts have stated that patients should be told about 'significant risks which would affect the judgement of a reasonable patient'. 'Significant' has not been legally defined, but the GMC requires doctors to tell patients about 'serious or frequently occurring' risks. In addition if patients make clear they have particular concerns about certain kinds of risk, you should make sure they are informed about these risks, even if they are very small or rare. You should always answer questions honestly. Sometimes, patients may make it clear that they do not want to have any information about the options, but want you to decide on their behalf. In such circumstances, you should do your best to ensure that the patient receives at least very basic information about what is proposed. Where information is refused, you should document this on page 2 of the form or in the patient's notes.
