# Supplementary material for: Patient-directed self-management of pain (PaDSMaP) compared to treatment as usual following total knee replacement; a randomised controlled trial
Source: BMC Health Serv Res. 2018 May 10;18:346. doi: 10.1186/s12913-018-3146-2 (PMC5944138; doi:10.1186/s12913-018-3146-2)
Supplement: Supplementary file 2 — Consent for Patients about to undergo Total Knee Replacement Surgery. (PDF 802 kb) [file 12913_2018_3146_MOESM2_ESM.pdf]

1. Information Sheet For Patients in the PaDSMaP Study
(Patient-Directed Self Management of Pain - PaDSMaP)

Part 1:

1.1 Title: Organisation of pain management after total knee replacement (PaDSMaP Study)

1.2. Invitation

We would like to invite you to take part in our research study. Before you decide we would like you to understand why the research is being done and what it would involve for you. **After you have had chance to read this, the research nurse will go through the information sheet with you and answer any questions you have.** This will be done when you attend for a pre-operative assessment approximately two weeks prior to your operation and will take at most 30 minutes. If you decide to take part in this research another 90 minutes of your time will be needed for assessments and education at the pre-assessment clinic (i.e. a total of 2 hours). You will also need to attend for an additional 2 hours at the follow-up clinic 6 weeks after the operation (see below for details). Talk to other people about the study if you wish. (Part 1 tells you the purpose of this study and what will happen to you if you take part. Part 2 gives you more detailed information about the conduct of the study). Ask us if there is anything that is not clear.

Thank you for reading this.

1.3. What is the research for?

You are invited to join in with some research about how to organise pain relief after a total knee replacement (TKR). We know that patients experienced significant pain after a TKR operation and this can increase the time taken to get back to walking and normal activities. Pain levels may be changed if the pain relief pills taken by mouth are taken by the patient when they want to take them (patient-directed self management of pain (PaDSMaP)) rather than when the nurse gives them as part of a drugs round in hospital (treatment as usual - TAU). The study will compare PaDSMaP with TAU in a research trial.

Sometimes we don‘t know which way of treating patients is best. To find out, we need to compare different treatments. We put people into groups and give each group a different treatment. The results are compared to see if one is better. To try to make sure the groups are the same to start with, each patient is put into a group by chance (randomly). The study will compare PaDSMaP with TAU in a research trial. You have an equal chance of being in either group. One hundred and forty-four patients will be randomly divided into the two groups. This study will investigate whether PaDSMaP improves levels of pain at three days after a TKR operation compared to TAU. It will also compare the two groups up to 6 weeks after the operation for satisfaction with control of pain, return to walking and normal activities, as well as any problems. We will also interview a few patients and ward staff to explore their experiences of the PaDSMaP and TAU protocols. Finally we will measure the costs of PaDSMaP.

It is hoped that this will allow the NHS to improve pain control for patients post-operatively after TKR. It is likely that the information from this study will be relevant to pain control in general after operations.

1.4. Why was I chosen?

You are about to undergo a total knee replacement operation for the first time at the Norfolk and Norwich University Hospital and you manage your own medications when you are at home.

1.5. Do I have to join in?

It is up to you to decide to join the study. We will describe the study and go through this information sheet. If you agree to take part, we will then ask you to sign a consent form. You will get a copy of both of these and should keep them safe so that you can read them at any time. You are free to withdraw at any time, without giving a reason. This would not affect the standard of care you receive.

1.6. What will happen to me if I take part?

If you agree to join in, you should sign the information sheet and consent form as explained above, and return these at the pre-operative assessment outpatient clinic (about 2-3 weeks before your operation). Keep a copy of each of these documents so that you can read them at any time.

The research nurse will then call the randomisation service at the University of East Anglia, who will check that you are eligible for this study and randomly put you in either the patient-directed self management of pain (PaDSMaP) group or the group where the nurse gives out the pain relief drugs as part of a drugs round in hospital (treatment as usual – TAU).

If you are put in the TAU group you will be asked to fill in questionnaires at this pre-operative assessment clinic, for the three days of your inpatient stay, and at your 6 week follow-up outpatient clinic. Otherwise your care will be as it would usually be.

If you are put in the PaDSMaP group the research nurse will give you some information on how to manage your pain relief, this will take about 20 minutes. This will also be provided in writing for you. You will be in charge of managing your pain relief taken by mouth whilst on the ward after your operation. You will also be asked to fill in questionnaires at this pre-operative clinic, for the three days of your inpatient stay, and at your 6 week follow-up outpatient clinic. Otherwise your care will be as it would usually be.

Details of what sort of questions you will be asked and when you will be asked them are outlined below.

Ten patients will also be asked to join in an interview with the research nurse to examine in more depth their views on the management of their post-op pain relief. If you are chosen to be one of these ten people we will give you some further information and a second consent form. As before, there is no obligation on you to participate and your care will not be affected.

1.6.a Questionnaires

You will be given a number of questionnaires to fill in. These will help us understand how much pain you are in at various times across the study. We will also measure items such as your satisfaction with your pain levels, your activities that may be affected by knee pain, and your general quality of life. It will also allow us to measure other things that may affect the amount of pain. These will include such things as the types and amounts of pain relief drugs you take, your mood, and whether the pain was better or worse than what you expected.

It is very important that you feel you can agree to fill in the questionnaires as fully as possible at all the times needed.

1.6.b Outpatient questionnaires

You will be given questionnaires to complete at your pre-operative assessment outpatient appointment (about 2-3 weeks prior to TKR) and at your post-operative outpatient appointment (about 6 weeks after TKR). These will be completed at both clinics except where stated.

- Pain level in knee
- Personal information questionnaire (such as age, other illnesses, length of time of knee pain) (this information will only be asked for once at the pre-operative assessment clinic only)
- Medication record (records all the pills you are on – for knee pain and all other pills)
- Oxford Knee Score (OKS - measures activities that may be affected by knee pain)
- Quality of life (QOL)
- Cost record (records health costs that are related to your knee pain e.g. GP visits)
- Hospital Anxiety and Depression Scale (HADS - measures mood and anxiety)
- Satisfaction with information questionnaire – measures how happy you are with the information provided about how to manage your post-operative pain (post-operative clinic only).

We expect the questionnaires will take 30-40 minutes to complete at each of these two clinics.

One of the questionnaires we will ask you to complete will assess your mood (HADS). If on completion we find that the questionnaire's result suggests that you are suffering from depression the research nurse will tell you immediately and give you an information sheet which will recommend what you should do next to help with this condition. We will also send a letter to your GP telling them that you have been assessed as being depressed.

1.6.c Inpatient questionnaires

These questionnaires will be started the day after your TKR operation once you are on pain relief pills that are to be taken by mouth.

The same questionnaires will be completed for each of the three days immediately after your operation whilst you remain in hospital. Should you be discharged earlier you will be asked to complete that day's set of questionnaires on satisfaction etc.

Questionnaires which need completing and when	Breakfast	Lunch	Supper	Just after physiotherapy
Pain score	✓	✓	✓	✓
Satisfaction with pain relief		✓		
Side effects		✓		
Pain relief pills/liquids	We will ask you to keep a record of each type of pill/liquid you take for pain, what dose, and at what time, each time you take any pain relief across the day.			

These inpatient questionnaires are all short and we expect that you would spend no more than 5 minutes at any given time to complete them.

1.7 Are there any risks to me?

It is possible that people in the PaDSMaP group could accidentally overdose on their medications. However everyone in this group will be provided with a clear timetable of which drugs should be taken and when. The research nurse, ward nurses and pharmacist will also be available to answer any questions a person has regarding their drugs. The staff will also check the amounts of drugs taken. Any overdose will be noted and managed appropriately.

1.8 Could joining in the study do me some good?

We currently do not know the best way to organise the delivery of oral pain relief to people after TKR. Either group could prove to be the better way to do this, or there could be no difference between the two groups.

You might also feel good about joining in research that tries to improve care for people about to undergo a TKR in the future, by helping doctors and nurses and other professionals know more about how best to organise the delivery of pain relief.

Both groups will receive good care from the healthcare team but the research may not help you personally more than the TKR would have done anyway.

1.9 Expenses and payments

Your hospital parking ticket will be validated by the research nurse.

1.10 What if there is a problem?

Any complaint about the way you have been dealt with during the study or any possible harm you might suffer will be addressed. The detailed information on this is given in Part 2.

1.11 Will my taking part in the study be kept confidential?

Yes. We will follow ethical and legal practice and all information about you will be handled in confidence. The details are included in Part 2.

Part 2:

2.1 What will happen if I start being involved in the study but then don’t want to carry on with the study?

You can stop being involved in the study at any time and you don’t have to give a reason.

If you are in the TAU group and withdraw from the study, you will then receive treatment as usual but without filling in any further questionnaires. But we will need to use the data collected up to your withdrawal.

If you are in the PaDSMaP group, you could withdraw from the PaDSMaP system and decide that you want the nurses to take over delivery of your medication (treatment as usual). You could keep on filling in the questionnaires (which would be very useful for us), or you could withdraw completely from the study. But again we will need to keep the data collected up to your withdrawal. You will not be able to continue self-administrating your medicines if you withdraw from the study.

2.2 What if there is a problem?

2.2.a Complaints

If you have a concern about any aspect of this study, you should ask to speak to the researchers who will do their best to answer your questions; Dr Katherine Deane, Project Lead Tel: 01603 597047, Monday to Friday, Office hours, E-mail: k.deane@uea.ac.uk or Prof Simon Donell, Principal Investigator, Tel: 01603 286706 Monday to Friday, Office hours, E-mail: simon.donell@nnuh.nhs.uk. The Patient Advice and Liaison Service, known as PALS, has been introduced to ensure that the NHS listens to patients, their relatives, carers and friends, and answers their questions and resolves their concerns as quickly as possible. PALS can be contacted by you to help resolve any concern you may have about any aspect of this study. They can be contacted Monday to Friday, Office hours, Tel: 01603 289036, Email Address: PALS@nnuh.nhs.uk
If you remain unhappy and wish to complain formally, you can do this by contacting the Complaints and Legal Services Department, Colney Lane, Norwich, NR4 7UY, Tel: 01603 289686 or 01603 289684. Further information on the Complaints Procedure may be found at www.nnuh.nhs.uk (click on 'Patient Info') or at www.dh.gov.uk.

2.2.b Harm

In the event that something does go wrong and you are harmed during the research and this is due to someone's negligence then you may have grounds for a legal action for compensation against the Norfolk and Norwich University Hospitals NHS Foundation Trust but you may have to pay your legal costs. The normal National Health Service complaints mechanisms will still be available to you (if appropriate).

2.3 Will my taking part in this study be kept confidential?

Yes. All information which is collected about you during the course of the research will be kept strictly confidential. All the information we obtain relating to you will be treated in the strictest confidence and stored in line with the Data Protection Act (1998). Only investigators from our team (who have formal legal duties of confidentiality) will have access to this information. Any information about you which leaves the hospital will have your details removed. We will give you a number when you join the study and this number will be used on all the documents instead of your name so you cannot be identified. With your agreement, we will write to your GP to tell them you are taking part in this trial.

Data from this research will be stored securely for up to five years after the study is completed to allow us to fully analyse all of the data. After this time the data will be disposed of securely.

2.4 What will happen to the results of the research study?

We will send you a summary of the results of the study and will try to publish results in research journals to do with health. Also, we will give the Arthritis Research Campaign a summary, to print in their newsletter if they wish.

The research team frequently speak at meetings about TKR surgery and medication management and will talk about the research in future meetings.

2.5 Who is organising and funding the research?

This study is funded by the National Institute for Health Research (NIHR) from the Research for Patient Benefit funding stream. It represents a collaboration between the Norfolk and Norwich University Hospital and the University of East Anglia.

2.6 Who has reviewed the study?

The study has been externally peer reviewed by experienced health researchers selected by NIHR. In addition all research in the NHS is looked at by independent group of people, called a Research Ethics Committee, to protect your interests. This study has been reviewed and given favourable opinion by Cambridgeshire 1 Research Ethics Committee. The East Norfolk and Waveney Research Governance Committee has reviewed its suitability to be run by an NHS hospital.

2.7 Contacts for Further Information

If you wish to discuss the study further or ask any questions about it, please contact:

Dr Katherine Deane, Project Lead
Tel: 01603 597047, Monday to Friday, Office hours
E-mail: k.deane@uea.ac.uk

Prof Simon Donell, Principal Investigator
Tel: 01603 286706, Monday to Friday, Office hours
E-mail: simon.donell@nnuh.nhs.uk

If you decide to take part, thank you for participating.
