# Supplementary material for: Patient-directed self-management of pain (PaDSMaP) compared to treatment as usual following total knee replacement; a randomised controlled trial
Source: BMC Health Serv Res. 2018 May 10;18:346. doi: 10.1186/s12913-018-3146-2 (PMC5944138; doi:10.1186/s12913-018-3146-2)
Supplement: Supplementary file 3 — Information Sheet for Patients in the PaDSMaP Study. (PDF 800 kb) [file 12913_2018_3146_MOESM3_ESM.pdf]

2. CONSENT FORM

For patients in the PaDSMaP study

(Patient-Directed Self Management of Pain - PaDSMaP)

Title of Project: Organisation of pain management after total knee replacement (PaDSMaP Study)

Name of Researcher:

	Please initial box
1. I confirm that I have read and understand the information sheet for the research project called '**Organisation of pain management after total knee replacement (PaDSMaP Study)**'.	☐
2. I have had a chance to think about it and ask any questions. I am sure that I know enough about it to help me decide about joining in.	☐
3. I know that I don't have to do this, It is my own choice. If I start joining in with the research I know that I can stop if want to at any time. I will still be cared for in the same way, whether I join in or not and I do not have to give a reason for stopping.	☐
4. I agree that the chief investigator (Prof Simon Donnell) should tell my GP about me taking part in the study, and should to tell my GP if there are any concerns about my health or welfare during the research.	☐
5. I agree to take part in the PaDSMaP research study.	☐

____________________	______________	______________________________
Name of Participant	Date	Signature
____________________	______________	______________________________
Name of Person taking consent	Date	Signature

Participant Identification Number (researcher to complete): _______________

When completed: 1 copy for participant; 1 copy for researcher site file; 1 (original) to be kept in medical notes.
