# Supplementary material for: Patient-directed self-management of pain (PaDSMaP) compared to treatment as usual following total knee replacement; a randomised controlled trial
Source: BMC Health Serv Res. 2018 May 10;18:346. doi: 10.1186/s12913-018-3146-2 (PMC5944138; doi:10.1186/s12913-018-3146-2)
Supplement: Supplementary file 4 — Consent form for Patients in the PaDSMaP Study. (PDF 741 kb) [file 12913_2018_3146_MOESM4_ESM.pdf]

Taking control of pain: Helping you to help yourself

Book 1: Thinking about pain management

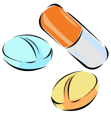

PaDSMaP

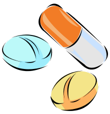

Self medicating patient information booklet for the Patient-Directed Self Management of Pain – the PaDSMaP study

Your clinical care team are:

- *PaDSMaP research nurse*
- *Nurses in outpatient clinics*
- *Ward nurses*
- *Ward pharmacists*
- *Physiotherapists*
- *Surgeons*
- *Anaesthetists*

Pain Points

1. Your clinical care team cannot promise you no pain but we will try to work with you to get as close to this goal as possible
2. Pain medicines work best if they are taken before the pain starts
3. Your clinical care team want you to ask questions even if they seem silly to you
4. Don't suffer in silence there is always something else we can try
5. Know your pain score i.e. mark it out of 10; and do something about it if your pain score is rising or your pain is more than a dull ache

How do I manage to control my pain?

The aim of this booklet is to help you control any pain you may feel after your total knee replacement. We aim to make sure that you experience the least possible post-operative pain, both immediately after the operation and when you go home. In our experience, planning your choices in advance will mean that you will get the pain control that works best for you. It really is worth putting the effort in now.

There are several treatments that can help control post-operative pain after a total knee replacement. They include medicines and additional non-medicinal treatments such as using ice packs on the knee, and relaxation techniques.

There are different types of medicine to consider. Often people will need a combination of medicines to control the pain. Post-operative pain is the sort of pain that responds well to medicines. But the amount and type of

People need medicines for pain after an operation:

- *To prevent pain*
- *To allow them to get mobile as soon as possible*
- *To prevent complications from not moving*
- *To prevent the development of*

medicines each person needs after an operation will vary.

Exercise 1: Thoughts about pain medicines

It is worth considering your personal experience of pain and controlling pain at this stage. Take 5 minutes to think about and write about this, as well as any worries or concerns you may have about your post-operative pain control. Write down the medicines or treatments that have been particularly helpful in treating the pain in your knee as well as those that have not been so helpful. It might also be helpful to think about what information you might like about your pain medicine choices. Look at the picture below and write your thoughts and ideas around the cloud as they occur to you. Try not to write a list.

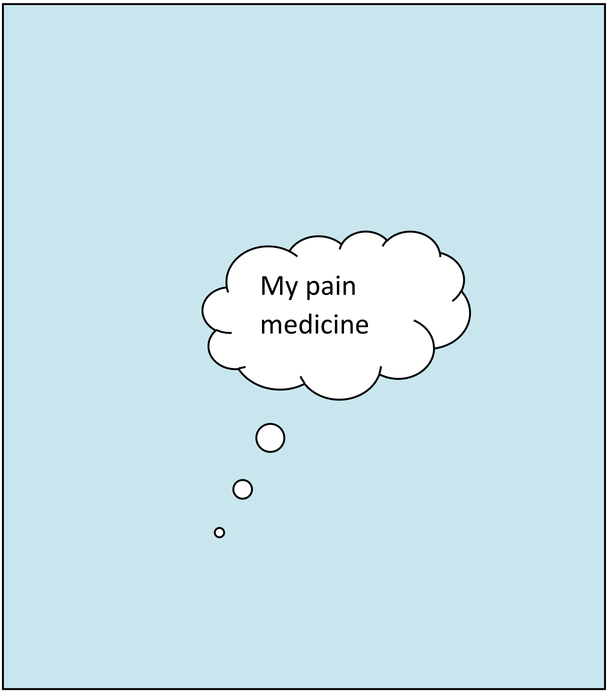

Exercise 2: My pain medicines

What medicine are you currently taking to control the pain in your knee? How helpful do you feel the medicine is at taking away the pain? What are your thoughts about taking pain medicines, both now and after the operation?

The following exercise will help you think about these issues.

Which medicine(s) are you currently taking to control the pain in your knee?

Name of medicine	How much (mg per day)
________________	___________
________________	___________
________________	___________
________________	___________
________________	___________
________________	___________
________________	___________

How helpful is your medicine(s) in controlling your pain?

1	2	3	4
Not Helpful	A bit helpful	Helpful	Very helpful

Exercise 3: Is pain medicine important for me?

What are your thoughts and feelings about your pain medicine? What do you think about your pain medicine? Do you think it is important? Do you think it is important to take after the operation?

How important do you think it is to take your pain medicine after the operation? Give yourself a rating out of ten.

1 2 3 4 5 6 7 8 9 10

Not important ← →Very important

Why do you think that?

____

____

____

____

If you have not rated taking your pain medicine after the operation as very important, what would have to happen for you think taking your pain medicine was more important?

____

____

____

Exercise 4: Exploring choices

Exercise 4 is a choice exercise that may help you think about the helpful and not so helpful aspects of the pain medicines you are currently taking. In particular are any of your pain medicines giving you problems (side-effects)? Remember the exercise is about your thoughts and feelings. There are no right or wrong answers.

The helpful things about taking this medicine	The unhelpful things about taking this medicine
________________	________________
________________	________________
________________	________________

Think about your answers? Where does this leave you now? Are these pain medicines right for you?

Completing Exercises 3 and 4 can sometimes uncover practical problems especially relating to the side-effects of medicine for pain.

You may wish to repeat Exercises 3 and 4 when you are actually taking the post-operative pain medicines (which may differ from your current ones), so there is a repeated set of these two exercises towards the back of this booklet.

My pain medicine, my choice

When weighing up how important taking my pain medicines are, I need to consider how helpful they are in taking the pain away and what side-effects they may also give me.

Getting the most from your doctor and nurse

Talking to doctors and nurses can be very difficult as conversations tend to be short. It can

be hard to assert yourself and express your concerns. The key to getting what you want out of the conversation is to be prepared. Think in advance about what your goal is for the conversation and try to stick to it.

Exercise 5: What do I need to know?

There may well be additional information that you want about controlling your post-operative pain after your total knee replacement.

Take 5 minutes to think about what you know and what you want to know about controlling your post-operative pain after your total knee replacement operation and the medicine you are taking or thinking of taking.

1. What do you currently know about post-operative pain control?

____

____

____

2. What do you currently know about your medicine choices for your post-operative pain control?

____

____

____

____

3. What additional information do you want about post-operative pain control?

____

____

____

____

4. What additional information do you want about pain control medicines?

____

____

____

____

You may find some of the answers to these questions in the second book: ***"Information about pain management".***

After you have given some thought to your goals and what you need to find out about, then you

need to think about the questions you want to ask. We have listed some ‘power questions’ that you might find useful when talking to your doctor or nurse.

Power questions for talking to doctors and nurses

‘Power questions’ are essential and focused questions you can ask during a meeting with a health professional. Having a set of questions can prepare you for the meeting so that you can get the most out of it. It can also help you feel more involved in the decisions about your care and treatment.

On the next pages there are a number of ‘power questions’ that might help focus your conversations. These are just suggestions – the list of the questions is repeated at the back of

this booklet so you can tear them out and take them to the meeting with you – and/or you may choose to come up with your own.

Before the meeting write down the questions you want answered and work through them one by one. For example, you could say to the health professional: “I have some questions about my medicine that are important to me. I was wondering if I could go through then with you?”

Examples of power questions

1. What choice of pain medicine is available to me for after the operation?

____

____

____

____

2. How do the pain medicines work?

____

____

____

____

3. How effective is the pain medicine for dealing with the post-operative pain?

____

____

____

____

4. What are the most common side-effects of these pain medicines?

____

____

____

____

5. What is the best way to prevent or deal with the side-effects I might get?

____

____

____

____

6. How long will I have to take the pain medicines to deal with the post-operative pain?

____

____

____

____

7. What will happen if/when I stop taking the pain medicine?

____

____

____

____

8. Do I need any special blood tests/health checks while I am taking the pain medicine?

____

____

____

____

9. How often will the pain medicine be reviewed?

____

____

____

____

10. If I choose not to take the pain medicine, what other treatment choices are available to me?

____

____

____

____

Contact details

We hope you have found this booklet useful and it has helped you think about the way your pain can be controlled and the type of things you can do to help.

Should you have any queries about the PaDSMaP study or managing your medicines ask the research nurse or the ward nurses and pharmacists in the first instance. If this does not resolve your query you can contact the researchers:

Dr Katherine Deane, Project Lead
Tel: 01603 597047, Monday to Friday, Office hours
E-mail: k.deane@uea.ac.uk

Prof Simon Donell, Principal Investigator
Tel: 01603 286706, Monday to Friday, Office hours
E-mail: simon.donell@nnuh.nhs.uk

It may be helpful to repeat exercises 3 & 4 after the operation, when you are actually taking the post-operative pain medicines.

Repeat of Exercise 3: Is post-operative pain medicine important for me?

What are your thoughts and feelings about your pain medicine? What do you think about your pain medicine? Do you think it is important? Do you think it is important to take after the operation?

How important do you think it is to take your pain medicine after the operation? Give yourself a rating out of ten.

1 2 3 4 5 6 7 8 9 10

Not important ← →Very important

Why do you think that?

____

____

____

____

If you have not rated taking your pain medicine after the operation as very important, what would have to happen for you think taking your pain medicine was more important?

____

____

Repeat of Exercise 4: Exploring choices post-operatively

Exercise 4 is a choice exercise that may help you think about the helpful and not so helpful aspects of the pain medicines you are currently taking. In particular are any of your pain medicines giving you problems (side-effects)? Remember the exercise is about your thoughts and feelings. There are no right or wrong answers.

The helpful things about taking this medicine	The unhelpful things about taking this medicine
______________________	____________________
______________________	____________________
______________________	____________________
______________________	____________________
______________________	____________________
______________________	____________________
______________________	____________________

Think about your answers? Where does this leave you now? Are these pain medicines right for you?

____

____

____

____

Completing Exercises 3 and 4 can sometimes uncover practical problems especially relating to the side-effects of medicine for pain.

Extra Set of Power Questions

1. What choice of pain medicine is available to me for after the operation?
2. How do the pain medicines work?
3. How effective is the pain medicine for dealing with the post-operative pain?
4. What are the most common side-effects of these pain medicines?
5. What is the best way to prevent or deal with the side-effects I might get?
6. How long will I have to take the pain medicines to deal with the post-operative pain?
7. What will happen if/when I stop taking the pain medicine?
8. Do I need any special blood tests/health checks while I am taking the pain medicine?
9. How often will the pain medicine be reviewed?
10. If I choose not to take the pain medicine, what other treatment choices are available to me?

Taking control of pain: Helping you to help yourself

Book 2: Information about pain management

PaDSMaP

Self medicating patient information booklet for the Patient-Directed Self Management of Pain – the PaDSMaP study

Your clinical care team are:

- *PaDSMaP research nurse*
- *Nurses in outpatient clinics*
- *Ward nurses*
- *Ward pharmacists*
- *Physiotherapists*
- *Surgeons*
- *Anaesthetists*

Pain Points

1. Your clinical care team cannot promise you no pain but we will try to work with you to get as close to this goal as possible
2. Pain medicines work best if they are taken before the pain starts
3. Your clinical care team want you to ask questions even if they seem silly to you
4. Don't suffer in silence there is always something else we can try
5. Know your pain score i.e. mark it out of 10; and do something about it if your pain score is rising or your pain is more than a dull ache

What do I need to know?

The information in this section may help *you* answer some of the questions about pain medicines you may have.

What can I expect after my total knee replacement operation?

After your operation your initial pain control will be pain medicines given to you by the nurses as tablets or injections. Some of you may be given pain control as a local anaesthetic into your joint (via a small tube into your knee joint – a catheter). You will be given physiotherapy soon after the operation, usually within 4 hours.

As soon as you are awake, alert and thinking clearly, you will be given access to your pain

medicines which will be stored in a locked box attached to your bed on the ward. You will have a key for this box. You can then take your pain medicines according to the Medicines Timetable you will be given and your pain levels. The pain medicine Oxycontine may be prescribed to some people after their operation. Oxycontine is the only pain medicine that is not in your box and will be given to you by a nurse in the morning and evening.

You will also be given regular physiotherapy (about three times a day, i.e. morning, afternoon and evening). The physiotherapist will be visiting other patients on the ward so we can’t be exact on times.

How does pain medicine work?

Pain medicine is, as the name suggests, medicine that works against pain. These medicines work by blocking the pain signals to the brain and some reduce the swelling (inflammation) at the operation site. There are a number of routes for pain signals which is why you may need to take a number of different types of pain medicines before you get good pain control.

Types of pain medicine

There are a number of types of pain medicine (analgesics). Pain medicines are organised into three groups.

- **Group one** – These are called non-opioid analgesics, examples of which include

paracetamol and ibuprofen. These pain medicines are used to control mild to moderate pain. They should be taken regularly, and preferably before pain starts, to work at their best. However it may be necessary to step up to the next group of pain medicines.

- **Group two** – these are called mild opioids, examples of which include codeine, and dihydrocodeine. Opiate medicines were originally made from opium poppies – hence the name. Nowadays they are usually made synthetically. They are used to control moderate pain. Group two pain medicines should be taken in addition to pain medicines from group one. Again to be most effective they should be taken regularly, and preferably before pain starts, to work at their best. However it may be necessary to step up to the next group of pain medicines.

• **Group three** – these are called strong opioids, examples of which include oramorph and oxycontin. They are used to control severe pain. Group three pain medicines should be taken in addition to pain medicines from group one and/or two. Again they should be taken regularly, and preferably before pain starts, to work at their best.

Pills and syrups

All of the pain medicines we will be discussing are taken by mouth (orally). Most are given as pills or tablets but a few are given as liquids or syrups.

> *Remember, all pain medicines work BEST if taken BEFORE the pain starts*

Pain medicines after the operation

Table 1 lists some of the pain medicines commonly given after your operation. To give you an idea of how much of a medicine you should be taking we have listed the typical amount of medicine that is given to people to control their pain after the operation. Different people need different doses of medicine; this is because everyone’s body deals with medicine differently. Even in the same person, the amount of medicine they need may vary according to what they are doing, and whether they are taking their medicine before or after pain has set in.

Table 1 shows the typical doses. Your doses may differ according to your levels of pain and what your body can tolerate e.g. ibuprofen can cause an upset stomach for some people. It is important that you get the right amount of the right medicines.

Table 1: Pain medicines

Name	Typical post-operative dose
**Paracetamol**	1000 mg/<br>4 times a day
**Ibuprofen**	400 mg/<br>3 times a day
**Gabapentin**	300 mg/<br>2 times a day
**Oxycontin***	10-20 mg/<br>2 times a day
**Oramorph**	5-20 mg as needed, up to every 2 hours

* Oxycontin is the only medicine on this list that will be given to you by the nurse in the morning and evening. All other medicines will be in your medicine locker for you to take.

Other medicines

If you usually take other medicines, for example for high blood pressure, these will also be in your medicine locker for you to take as usual.

It is important that you get the right amount of the right medicines at the right time.

If you are ever worried about taking your medicines please ask someone from the clinical care team for advice.

Different amounts of pain medicine

You will notice that different amounts of each medicine are recommended (for example 1000 mg four times a day of paracetamol compared to 200 mg three times a day of ibuprofen). This is to do with how the medicine works.

Long and short acting pain medicine

Pain medicines can also work for different times, some are relatively short acting and so have to be taken regularly e.g. paracetamol four times a day, whilst others work over a longer time and so are taken less often e.g. gabapentin is only taken twice a day.

Safe doses of pain medicine

Finally some drugs have very tight dose ranges in which they are safe and effective e.g. paracetamol should never be taken more than four times a day, but other drugs have a greater range e.g. you may be prescribed 800mg ibuprofen a day (twice the usual dose) and this is entirely safe. However you must never take more medicine than is prescribed for you. If you are worried you don't have enough medicine to control your pain please talk to your clinical care team.

Because it can be quite complex to know what drug you should be taking and when, you will be provided with your own personal Medicine Timetable similar to the one overleaf.

Table 2: An Example of a Medicines Timetable

Time	Medicines to be taken
**06:00**	Paracetamol, Oxycontin*
**07:00**	
**08:00**	Gabapentin, Ibuprofen (after breakfast),
**09:00**	
**10:00**	Paracetamol
**11:00**	
**12:00**	Ibuprofen (after lunch)
**13:00**	
**14:00**	Paracetamol
**15:00**	
**16:00**	Ibuprofen (after tea)
**17:00**	
**18:00**	Paracetamol, Oxycontin*
**19:00**	
**20:00**	Gabapentin

N.B. If you cannot tolerate ibuprofen you will not have this medicine on your timetable. Ibuprofen should always be taken after food.

Oxycontin* will be given to you by the nurse.

The medicines don't work (breakthrough pain)

If you are in pain, first check your pain medicine timetable and make sure you have taken all the pain medicines you are supposed to have. If you have missed a dose of medicine take that dose immediately. Please remember that it can take 30-60 minutes for the pain medicines to start working. If you are unsure what you should take next after having missed a dose please ask a nurse for advice.

Oramorph works pretty quickly (in about 15 minutes) so it is very good for controlling pain that "breaks through" after taking the medicines

on your timetable. 20mg of Oramorph can be taken up to every two hours but we would usually expect you to need 2 or 3 doses on the first day after your operation (so usually taken at breakfast, lunch and supper times), and maybe 2 doses on the second day (usually taken at breakfast and supper times), and we wouldn’t usually expect you to need any doses of Oramorph on the third day after your operation. If you find that you need more doses of Oramorph than described above your clinical team will review your other pain medicines.

If you have taken all of the medicines you should have and are still in pain then immediately speak to a nurse about other pain medicines you could take to control your breakthrough pain.

Why do I need to control my pain?

OK, so realistically we cannot promise you no pain. But our job is to get as close to that goal as possible. And post-operative pain is the sort of pain that responds very well to pain medicines.

If you are in pain you are less likely to move and do your physiotherapy. This can increase your risk of developing blood clots in your veins (deep vein thrombosis or DVTs) which are a serious complication and may mean that you have to remain in hospital for longer. Also if you are not in pain, you can move your knee to a greater extent and this will mean that we can get you home sooner.

People need medicines for pain after an operation:

- *To prevent pain*
- *To allow them to get mobile as soon as possible*
- *To prevent complications from not moving*
- *To prevent the development of long term pain problems*
- To allow them to return home as soon as possible

Scientists have found that the brain can learn to stay in pain after the area that was damaged (e.g. in an operation) has healed. These long-term pain problems are not easy to treat and it is far better to prevent them starting. The best way to prevent such a long-term pain problem starting is to keep on top of the pain after the operation

and keep the pain levels as low as possible until you have fully recovered.

When to take pain medicines

Pain medicines work best when they are taken BEFORE the pain is felt. If taken after pain has set in, it usually requires more medicine to control the pain. And bear in mind that it can take 30-60 minutes for the pain medicines to start working (a long time to be in unnecessary pain). We know that pain can be best controlled when medicines are taken “by the clock” i.e. by following your timetable. However any breakthrough pain (i.e. pain that occurs between your timetabled doses of medicine) should also be treated promptly and effectively. So careful monitoring of pain levels is a key rule of pain management, and hourly monitoring of pain levels is encouraged. Finally if you are about to do something - like physiotherapy – that is likely to worsen the pain

in your knee, then you may wish to take an additional dose of pain medicine 15-30 minutes in advance of the event so that you control the pain and maximise your ability to do the physiotherapy without too much pain.

So, control of your pain after the operation starts with a "by the clock" approach to taking your pain medicines (to prevent pain before it resurfaces), ongoing monitoring of your pain levels to ensure there is no breakthrough pain, and then either additional "as needed" doses or a reduction in the amount of pain medicines to be taken depending on pain levels.

Pain Rules

- *Don't grin and bear it*
- *Take your medicines on time*
- *Keep an eye on your pain levels and do something about rising pain levels or anything above a dull ache*

Physiotherapy and pain

Physiotherapists need to get your knee moving so it heals quicker and better. This movement of your knee can be painful.

Oramorph may be particularly useful for controlling the pain that comes with doing your physiotherapy. If when the physiotherapist visits your bed to do the exercises your knee feel too sore to do them, you can ask them to visit someone else first and then take a dose of Oramorph. By the time the physiotherapist returns the pain should be reduced enough for you to do your exercises. Of course, if you didn't take a dose beforehand but your knee feels sore after physiotherapy, you can take a dose of Oramorph then. Remember the aim is to be in as little pain as possible. Do take care not to go over your total dose of Oramorph for a day - this will be written on your Inpatient Prescription Chart.

Jane's physiotherapy story

Jane was expecting to see the physiotherapist about 11am. So at 10am she scored her pain as being a bit sore (which she rated as 3 out of 10). She took her paracetamol then but decided that she didn't need any Oramorph. When the physiotherapist arrived at 11am Jane was in very little pain (score of 1) but the last set of exercises had make her knee quite sore and painful (score of 7 afterwards). She asked the physiotherapist if they could see someone else for now so she could take her Oramorph. She decided that 10mg of Oramorph would be enough (half the dose she could have taken). 15 minutes later the physiotherapist returned and Jane was able to do the exercises well without too sharp a pain. However afterwards her knee was throbbing and painful (score of 5) so she used an ice pack for 20 minutes and took the rest of the Oramorph (10mg) she was "allowed" for the 2 hour 10-12am time slot. This dropped her pain after a short while to a dull ache (score of 2) which she was quite happy with until her 12 noon doses of pain medicines.

How long do I need to take the pain medicines for?

Group 3 medicines (Oxycontine and Oramorph) are usually only given whilst you are still on the ward in the hospital. You will usually be given some Group 1 and 2 pain medicines to take home with you. However you must visit your GP for a prescription for more pain medicines. You will be given instructions on what medicines to take and when. Usually by the time you return for the post-operative check-up clinic (about 6 weeks after the operation) you will not be taking any pain medicines for your knee.

A rough guide to the side effects of pain medicines

No effective medicine is side effect free and pain medicines are no different. They can cause a

number of side effects that can be unpleasant and difficult to live with.

Table 3: Ways of sorting out side effects

Common Side effects	What can be done
**Feeling or being sick**	• If this is after ibuprofen or gabapentininform the nurse and your medicine will probably be changed<br>• If this is after opiates inform the nurse and you can be given anti-sickness pills
**Constipation**	• Constipation is easier to prevent than treat<br>• Try to prevent constipation by eating a high fibre diet (e.g. you can bring in some high fibre food with you to the ward such as bran flakes and dried fruits) and having plenty of drinks before and after your operation.

	• Try to prevent constipation by trying to become active again as soon as possible after the operation<br>• If you do become constipated ask the nurse for a laxative<br>• If you have had previous problems with constipation after pain medicines, you will be given some laxatives to take after the operation.
**Drowsiness**	• If you feel unusually drowsy after taking pain medicines let the nurse know and your medicines will be reviewed.
**Itching**	• Let the nurse know and you can be given antihistamines which will usually reduce this

Other concerns about taking pain medicines

Some patients are concerned that if they start taking strong pain killers then they may have difficulty stopping taking them. In fact you don't get addicted to opiates taken for short term pain

such as post-operative pain. The plan for pain medicines after your operation anticipates that the pain will lessen over the days, and the amounts and types of pain medicines are reduced alongside this. So you will only be taking the strong (addictive) medicines for a short period of time. This means that you are very unlikely to develop any problems with addiction.

Recording the medicines you take

You will be asked to record all the medicines you take on the Inpatient Prescription Chart that is kept at your bedside. Here you need to note which medicine you took, what dose, and exactly what time you took it at. There is a separate booklet showing you how to fill this in step by step.

If you have any concerns about how to record your medicines on the form the research nurse or ward nurses will be happy to explain this to you.

Safety of your medicines whilst on the ward

You will have a locker with your pain medicines in it which will be attached to your bed. It will also contain any other medicines you usually take. The locker will have a lock on it. Please make sure that other than when you are getting your medicines out your locker is kept locked. Be particularly careful of this if you have any children visiting. The medicines are prescribed just for you and should not be given to anyone else under any circumstances.

Contact details

We hope you have found this booklet useful and it has helped you think about the way your pain can be controlled and the type of things you can do to help.

Should you have any queries about the PaDSMaP study or managing your medicines ask the research nurse or the ward nurses and pharmacists in the first instance. If this does not resolve your query you can contact the researchers:

Dr Katherine Deane, Project Lead
Tel: 01603 597047, Monday to Friday, Office hours
E-mail: k.deane@uea.ac.uk

Prof Simon Donell, Principal Investigator
Tel: 01603 286706, Monday to Friday, Office hours
E-mail: simon.donell@nnuh.nhs.uk

Taking control of pain: Helping you to help yourself

Book 3: Self Help Tools

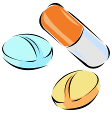 PaDSMaP 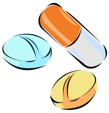

Self medicating patient information booklet for the Patient-Directed Self Management of Pain – the PaDSMaP study

Your clinical care team are:

- *PaDSMaP research nurse*
- *Nurses in outpatient clinics*
- *Ward nurses*
- *Ward pharmacists*
- *Physiotherapists*
- *Surgeons*
- *Anaesthetists*

Pain Points

1. **Your clinical care team cannot promise you no pain but we will try to work with you to get as close to this goal as possible**
2. **Pain medicines work best if they are taken before the pain starts**
3. **Your clinical care team want you to ask questions even if they seem silly to you**
4. **Don't suffer in silence there is always something else we can try**
5. **Know your pain score i.e. mark it out of 10; and do something about it if your pain score is rising or your pain is more than a dull ache**

People need medicines for pain after an operation:

- *To prevent pain*
- *To allow them to get mobile as soon as possible*
- *To prevent complications from not moving*
- *To prevent the development of long term pain problems*
- *To allow them to return home as soon as possible*

Making choices in relation to pain levels

People's level of pain can get worse for lots of reasons. If you regularly note your pain score – every hour – then this ensures that pain levels have not crept up on you unnoticed. It can be helpful to have made choices in advance about how to manage your pain. We will call this plan Advanced Pain Choices. The accompanying form

is an example of an Advanced Pain Choice that you might want to use.

Advanced Pain Choices: Pain diary

Give yourself a rating of your current pain level out of ten. With 10 being the worst possible pain and zero being no pain.

Do something about your pain levels (i.e. take some medicine) if your pain score is rising or your pain is more than a dull ache

Remember, all pain medicines work BEST if taken BEFORE the pain starts

Pain Score Diary

Worst pain	10																								
	9																								
	8																								
	7																								
	6																								
	5																								
	4																								
	3																								
	2																								
	1																								
No pain	0																								
Hours		1	2	3	4	5	6	7	8	9	10	11	12	13	14	15	16	17	18	19	20	21	22	23	24

Additional pain control techniques

We would recommend that these techniques are used in addition to the pain medicines. You can use them on the ward and when you go home. They are helpful to enhance pain medicines but should not be regarded as a replacement for them as they are not as effective as pain medicines. Therefore we cannot recommend them alone to control your pain effectively.

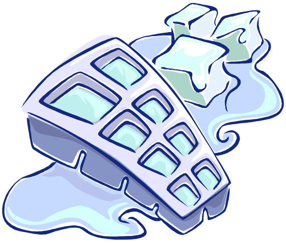

Using ice packs on the knee

Using ice on the knee will reduce pain and swelling. Apply ice or cold packs immediately to prevent or minimise swelling. Apply the ice or cold pack for 10 to 20 minutes, three or more times a day.

Swelling in the knee soon after your total knee replacement operation is common. The knee become painful and tender, both directly from the operation and indirectly from the swelling afterwards. This leads to the stiffness, pain, and tenderness.

An ice pack will help by reducing the swelling and also gives some pain relief. They are particularly recommended for immediately after physiotherapy sessions or other periods when you have had to use your knee more.

Can you apply too much ice?

Yes. An easy way to properly apply ice is to use crushed ice in a plastic bag covered with a moistened towel. This way the ice easily moulds itself around your knee. Alternatively, a bag of frozen vegetables can be used in the same way. The cold pack should be applied over a moist towel on the injured body part, do not put the pack directly onto the area which could cause damage to the skin. Apply for 20 minutes and remove for at least 20 to 40 minutes so that the skin is not injured from

the ice. The doctor, nurse or physiotherapist will be able to advise you if you have any questions about when or how to use an ice pack.

Relaxation

Progressive Muscle Relaxation (PMR) is a great technique for reducing overall body tension. It can also aid pain control as tension tends to increase pain. Here's how to get started:

1. Find several free minutes to practice progressive muscle relaxation, sit or lie down and make yourself comfortable.

2. Begin by tensing all the muscles in your face. Make a tight grimace, close your eyes as tightly as possible, clench your teeth, even move your ears up if you

can. Hold this for the count of eight as you inhale.

3. Now exhale and relax completely. Let your face go completely lax, as though you were sleeping. Feel the tension seep from your facial muscles, and enjoy the feeling.

4. Next, completely tense your neck and shoulders, again inhaling and counting to eight. Then exhale and relax.

5. Continue down your body, repeating the procedure with the following muscle groups:
    - chest
    - abdomen
    - entire right arm
    - right forearm and hand (making a fist)
    - right hand
    - entire left arm

- left forearm and hand (again, making a fist)
- left hand
- buttocks
- entire right leg
- lower right leg and foot
- right foot
- entire left leg
- lower left leg and foot
- left foot

6. For the shortened version, which includes just four main muscle groups:
    - face
    - neck, shoulders and arms
    - abdomen and chest
    - buttocks, legs and feet

Quickly focusing on each group one after the other, with practice you can relax your body like ‘liquid relaxation’ poured on your head and it flowed down and completely covered you. You can use

progressive muscle relaxation to quickly de-stress any time.

Breathing exercises

Breathing exercises are an ideal way to relieve stress in that they're fast, simple, free, and can be performed by just about anyone. Again reducing stress can reduce pain levels. Here's how basic controlled breathing works:

1. Sit or stand in a relaxed position.

2. Slowly inhale through your nose, counting to five in your head.

3. Let the air out from your mouth, counting to eight in your head as it

leaves your lungs. Repeat several times. That's it!

Tips:

1. As you breathe, let your abdomen expand outward, rather than raising your shoulders. This is a more relaxed and natural way to breathe, and helps your lungs fill themselves more fully with fresh air, releasing more "old" air.

2. You can do this just a few times to release tension, or for several minutes as a form of meditation.

3. If you like, you can make your throat a little tighter as you exhale so the air comes out like a whisper. This type of breathing is used in some forms of yoga and can add additional tension relief.

Contact details

We hope you have found this booklet useful and it has helped you think about the way your pain can be controlled and the type of things you can do to help.

Should you have any queries about the PaDSMaP study or managing your medicines ask the research nurse or the ward nurses and pharmacists in the first instance. If this does not resolve your query you can contact the researchers:

Dr Katherine Deane, Project Lead
Tel: 01603 597047, Monday to Friday, Office hours
E-mail: k.deane@uea.ac.uk

Prof Simon Donell, Principal Investigator
Tel: 01603 286706, Monday to Friday,
Office hours
E-mail: simon.donell@nnuh.nhs.uk

Taking control of pain: Helping you to help yourself

Book 4: Patient's stories

PaDSMaP

Self medicating patient information booklet for the Patient-Directed Self Management of Pain – the PaDSMaP study

Your clinical care team are:

- *PaDSMaP research nurse*
- *Nurses in outpatient clinics*
- *Ward nurses*
- *Ward pharmacists*
- *Physiotherapists*
- *Surgeons*
- *Anaesthetists*

Pain Points

1. Your clinical care team cannot promise you no pain but we will try to work with you to get as close to this goal as possible
2. Pain medicines work best if they are taken before the pain starts
3. Your clinical care team want you to ask questions even if they seem silly to you
4. Don't suffer in silence there is always something else we can try
5. Know your pain score i.e. mark it out of 10; and do something about it if your pain score is rising or your pain is more than a dull ache

Patient's stories

The aim of this booklet is to give you some examples of how people have applied the information we have discussed in the previous three booklets.

We hope that by placing the information in more "real-world" settings, you will be able to see how you can apply all of the information to your particular situation.

Tom's day after the operation

Yesterday Tom had his knee operation. He recovered well and is now in control of his pain medicines.

He woke at 6am in quite a lot of pain (7 out of 10 on the pain scale), so immediately looked at his drug timetable and saw he could take Paracetamol and Oramorph.

These helped but when he did his pain score again at 7am he was still in some pain (pain score of 5) so decided to take his Ibuprofen an hour early with a small snack (it’s best not taken on an empty stomach).

By the time the ward drug round came at 8am he was still in some pain (pain score of 4) but this eased within 30 minutes of taking the Gabapentin and being given the Oxycontine by the ward nurse, and dropped to a pain score of 1.

The physiotherapist came to Tom at 10am, but he asked if she could come back in just a little while as he hadn’t taken his 10am pain medicines yet. Then Tom took his Paracetamol and Oramorph, and the physiotherapist returned in about 15 minutes, by which time Tom was a lot happier about doing his physiotherapy. The physiotherapy did make his knee sore (pain score of 4) and swollen so Tom put

an ice pack on it for 20 minutes which reduced the swelling. He also did some relaxation techniques afterwards, and by 11am his pain score was good again (pain score of 1).

Tom took his ibuprofen immediately after his lunch but as his pain score was low he did not need to take any more Oramorph. After his second dose of physiotherapy for the day he put another ice pack on his knee but the pain reduced quicker this time, so at 2pm he only took some paracetamol.

At 4pm Tom had just taken his Ibuprofen and soon after the physiotherapists arrived. Tom found this third session of physiotherapy a bit sorer (pain score of 5). However with another ice pack and some relaxation afterwards he found that the pain soon reduced.

Knowing that he wasn’t to have any more physiotherapy Tom felt he only needed his final Paracetamol with his supper at 6pm.

Later in the evening (8pm), Tom felt his knee had stiffened up a bit and was rather painful after a trip to the toilet (pain score of 4). He took his longer acting pain medicines (Oxycontin (given by the ward nurse) and Gabapentin) and his pain score soon dropped to a score of 1.

Finally at 10pm Tom took a final dose of Oramorph as he was turning in for the night, because although his pain score was low he wanted to make sure he got a good night’s sleep. Using the relaxation exercises meant that he drifted off to sleep easily.

Tom's day

Time	Activity	Pain score	Drug timetable	When drugs were actually taken (comments)	Non-drug pain control
**06:00**	Wake	7/10	Paracetamol	06:00 Paracetamol, Oramorph*	
**07:00**	Breakfast	5/10		07:10 Ibuprofen (taken early as pain score high)	
**08:00**	Ward drug round	4/10	[Oxycontine (oxycodone)], Gabapentin (Neurontin), iburprofen	[08:15 Oxycontine (oxycodone) given by nurse], 08:00 Gabapentin (Neurontin)	
**09:00**		1/10			
**10:00**	Physiotherapy	4/10	Paracetamol	10:05 Paracetamol, Oramorph (took drugs about 15 min before doing physiotherapy)	Ice after physiotherapy
**11:00**		2/10			Relaxation
**12:00**	Lunch	1/10	Ibuprofen	12:10 Ibuprofen	
**13:00**	Physiotherapy	4/10			Ice after physiotherapy
**14:00**		2/10	Paracetamol	14:00 Paracetamol	Relaxation
**15:00**		1/10			
**16:00**	Physiotherapy	5/10	Ibuprofen	16:00 Ibuprofen	Ice after physiotherapy
**17:00**		2/10			Relaxation
**18:00**	Supper	1/10	Paracetamol	18:05 Paracetamol	
**19:00**		2/10			
**20:00**	Ward drug round	4/10	[Oxycontine (oxycodone)], Gabapentin (Neurontin)	[20:10 Oxycontine (oxycodone) given by nurse], 20:00 Gabapentin (Neurontin)	
**21:00**		1/10			
**22:00**	Sleep	1/10	Oramorph	22:05 Oramorph (took this to ensure good night's sleep)	Relaxation

*Oramorph can be taken variably – up to 20mg every two hours. Oxycontine is also known as Oxycodone; Gabapentin is also known as Neurontin.

Tom’s Pain Score Diary: First Day after Operation

Worst pain	10																								
	9																								
	8																								
	7						X																		
	6																								
	5							X									X								
	4								X		X			X							X				
	3																								
	2											X			X			X		X					
	1									X			X			X			X			X	X		
No pain	0																								
		1	2	3	4	5	6	7	8	9	10	11	12	13	14	15	16	17	18	19	20	21	22	23	24

Hour

Jane's physiotherapy story

Jane was expecting to see the physiotherapist about 11am. So at 10am she scored her pain as being a bit sore (which she rated as 3 out of 10). She took her paracetamol then but decided that she didn't need any Oramorph. When the physiotherapist arrived at 11am Jane was in very little pain (score of 1) but the last set of exercises had make her knee quite sore and painful (score of 7 afterwards). She asked the physiotherapist if they could go and see someone else for now so she could take her Oramorph. She decided that 10mg of Oramorph would be enough (half the dose she could have taken). 15 minutes later the physiotherapist returned and Jane was able to do the exercises well without too sharp a pain. However afterwards her knee was throbbing and painful (score of 5) so she

used an ice pack for 20 minutes and took the rest of the Oramorph (10mg) she was "allowed" for the 2 hour 10-12am time slot. This dropped her pain after a short while to a dull ache (score of 2) which she was quite happy with until her 12 noon doses of pain medicines.

Mary's story

Mary didn't like taking medicines, but as her knee had become more and more painful in the months before her operation she found that she sometimes had to "give in" and take a few paracetamol.

Mary was quite put off by the long list of pain medicines it seemed she ought to take after the operation. She had been put

in the self management of pain medicines arm of the PaDSMaP study. So she decided that she would try to manage with as few medicines as possible after the operation.

The day after the operation Mary's knee was very painful (pain score of 5) and she didn't want to move very much. In the morning when the physiotherapist tried to help her from the bed onto a chair next to it she felt so dizzy with pain they had to give up initially. But the physiotherapist came back again at lunchtime, and having seen that Mary had only taken paracetamol that day for her pain, asked her to rate her pain out of 10. Mary didn't want to be a bother so said it was 3 out of 10, although really she felt it had gotten more swollen and stiff since the morning and she really dreaded moving it.

The physiotherapist persuaded Mary to try and take some Oramorph for her pain, and came back to her after 30 minutes. By that time Mary was in less pain (pain score of 2) and with the physiotherapists help she managed to get to sit in her chair.

The research nurse then came and sat with Mary and asked if she had any worries about taking the pain medicines. Mary confided that she hated the idea of becoming dependent on pills. The research nurse reassured her that because she would only take the very strong pain killers for a short time she wouldn't become dependent. She then went through Mary's Pain Medicine Timetable and helped her sort out which medicines she should take next.

Mary found that by following her Timetable from then on she was never in as much pain as on the first morning. She was able to move more easily for the physiotherapy and was delighted to get home half a day early.

Contact details

We hope you have found this booklet useful and it has helped you think about the way your pain can be controlled and the type of things you can do to help.

Should you have any queries about the PaDSMaP study or managing your medicines ask the research nurse or the ward nurses and pharmacists in the first instance. If this does not resolve your query you can contact the researchers:

Dr Katherine Deane, Project Lead
Tel: 01603 597047, Monday to Friday,
Office hours
E-mail: k.deane@uea.ac.uk

Prof Simon Donell, Principal Investigator
Tel: 01603 286706, Monday to Friday,

Office hours
E-mail: simon.donell@nnuh.nhs.uk
